# In Vitro Anticancer Potential of *Jasione montana* and Its Main Components against Human Amelanotic Melanoma Cells

**DOI:** 10.3390/ijms22073345

**Published:** 2021-03-25

**Authors:** Aleksandra Maria Juszczak, Robert Czarnomysy, Jakub Władysław Strawa, Marijana Zovko Končić, Krzysztof Bielawski, Michał Tomczyk

**Affiliations:** 1 Department of Pharmacognosy, Faculty of Pharmacy with the Division of Laboratory Medicine, Medical University of Białystok, ul. Mickiewicza 2a, 15-230 Białystok, Poland; aleksandra.juszczak@umb.edu.pl (A.M.J.); jakub.strawa@umb.edu.pl (J.W.S.); 2 Department of Synthesis and Technology of Drugs, Faculty of Pharmacy with the Division of Laboratory Medicine, Medical University of Białystok, ul. Kilińskiego 1, 15-089 Białystok, Poland; robert.czarnomysy@umb.edu.pl (R.C.); kbiel@umb.edu.pl (K.B.); 3 Department of Pharmacognosy, Faculty of Pharmacy and Biochemistry, University of Zagreb, Marulićev trg 20/II, 10000 Zagreb, Croatia; mzovko@pharma.hr

**Keywords:** *Jasione montana*, Campanulaceae, flavonoids, luteolin derivatives, fibroblasts, melanoma

## Abstract

*Jasione montana* L. (Campanulaceae) is used in traditional Belarusian herbal medicine for sleep disorders in children, but the chemical composition and biological activity have not been investigated. In this study, the activities of *J. montana* extracts, their fractions and main compounds were evaluated in amelanotic melanoma C32 (CRL-1585) cells and normal fibroblasts (PCS-201-012). The extracts and fractions were analyzed using liquid chromatography–photodiode array detection–electrospray ionization–mass spectrometry (LC–PDA–ESI–MS/TOF) to characterize 25 compounds. Further, three major and known constituents, luteolin (**22**) and its derivatives such as 7-*O*-glucoside (**12**) and 7-*O*-sambubioside (**9**) were isolated and identified. The cytotoxic activities against fibroblasts and the amelanotic melanoma cell line were determined using the fixable viability stain (FVS) assay. The influence of diethyl ether (Et_2_O) fraction (**JM4**) and **22** on apoptosis induction was investigated using an annexin V binding assay. The obtained results showed significant cytotoxicity of **JM4** and **22** with IC_50_ values of 119.7 ± 3.2 and 95.1 ± 7.2 μg/mL, respectively. The proapoptotic potential after **22** treatment in the C32 human amelanotic melanoma cell line was comparable to that of vinblastine sulfate (VLB), detecting 29.2 ± 3.0% apoptotic cells. Moreover, **22** displayed less necrotic potential against melanoma cells than VLB. In addition, the influences of **JM4** and **22** on the dysfunction of the mitochondrial membrane potential (MMP), cell cycle and activity of caspases 3, 8, 9, and 10 were established. The effects of **JM4** on MMP change (74.5 ± 3.0% of the cells showed a reduced MMP) corresponded to the results obtained from the annexin V binding assay and activation of caspase-9. **JM4** and **22** displayed a significant impact on caspase-9 (40.9 ± 2.4% of the cells contained active caspase-9 after **JM4** treatment and 16.6 ± 0.8% after incubation with **22**) and the intrinsic (mitochondrial) apoptotic pathway. Moreover, studies have shown that **JM4** and **22** affect the activation of external apoptosis pathways by inducing the caspase-8 and caspase-10 cascades. Thus, activation of caspase-3 and DNA damage via external and internal apoptotic pathways were observed after treatment with **JM4** and **22**. The obtained results suggest that *J. montana* extracts could be developed as new topical preparations with potential anticancer properties due to their promising cytotoxic and proapoptotic potential.

## 1. Introduction

Skin cancers are the most common type of human cancer, with dramatically increasing incidence and mortality rates. The World Health Organization (WHO, Geneva, Switzerland) reports that globally, approximately 3 million skin cancers occur annually, of which less than 6% are melanomas [1]. Cutaneous malignant melanoma (CMM) developed by transformed melanocytes proliferating from the basal area of the epidermis, is the most aggressive form of diagnosed skin cancer [2,3]. Even though the incidence of melanoma is relatively low, its mortality is the highest among all skin cancers [4]. Although several new drugs have been developed over the last 10 years that have greatly improved the prognosis of patients with metastatic melanoma, many patients do not show a lasting response to these treatments [5]. Treatment with natural compounds and preparations based on plant materials have been increasingly used among patients with various neoplasms [6,7,8], and natural product-based treatments for melanoma are constantly being researched [9,10].

*Jasione montana* L., commonly known as Sheep’s bit scabious, is one of 16 representatives of the genus *Jasione* L. (Campanulaceae). The presence of *J. montana* outside of Europe, the Scandinavian Peninsula, Great Britain, and Ireland has been established, inter alia, in Morocco, Tunisia, Algeria, Russia, and Turkey, as well as in the northeastern region of the United States. It is a biennial plant, or occasionally, an annual plant, mainly found in warmer, sunny or semiarid places in lowland and upland regions. The determining factors for the distribution of *J. montana* are the temperature and average precipitation per year [11,12]. *J. montana* has been recognized as a garden plant [11]. Ethnopharmacological reports regarding this traditional Belarusian herbal medicine state that *J. montana* is used to treat sleep disorders in children, although its biological activity has not yet been assessed [13]. The available scientific literature on *J. montana* is scarce, and little is known about its phytochemical composition. A previous study of the aerial parts of *J. montana* established the presence of the bioactive flavonoid luteolin [14]. Taking these aspects into consideration, a detailed phytochemical analysis and characterization of the *J. montana* aerial parts was undertaken. Furthermore, the activities of the extracts, their fractions and isolated compounds against a human amelanotic melanoma cell line were investigated.

## 2. Results

### 2.1. LC–ESI–MS Analysis of Extracts **JM1**–**JM3** and Fractions **JM4**–**JM6**

The phytochemical analysis, based on liquid chromatography–photodiode array detection–electrospray ionization–mass spectrometry (LC–PDA–ESI–MS/TOF) technique, results of the extracts (H_2_O, **JM1**; 50% MeOH, **JM2**; MeOH, **JM3**), and their fractions (Et_2_O, **JM4**; EtOAc, **JM5**; *n*-BuOH, **JM6**) revealed 25 polyphenolic compounds. Extraction of the raw material with MeOH (**JM3**) resulted in a high content of free aglycones [peaks **22–25**], among which luteolin (**22**) was the dominant peak. The second most represented compound was luteolin 7-*O*-glucoside (**12**). On the other hand, **JM1** and **JM2** were rich in glycosides, and the hydromethanolic extractant was the more efficient of the two solutions. The studied fractions (**JM4**-**JM6**) were characterized by the selectivity of the extraction process for specific groups of compounds. **JM4** turned out to be rich in aglycones with a dominance of **22** as well as apigenin (**23**) and **12**. Moreover, *p*-coumaric acid and its derivatives were also present in this faction [peaks **3**, **10**]. An absorption maximum at 310 nm in the ultraviolet-visible (UV-VIS) spectrum is characteristic for these types of compounds. Compound **12** was also significantly dominated in the **JM5** fraction. The presence of di- and triglycosides was also revealed [peaks **9**, **14**, **15**]. These compound structures were suggested based on the loss of fragments [M − 162 ± H]^+/−^, [M − 146 ± H]^+/−^, and [M − 132 ± H]^+/−^, which correspond to *O*-hexose, *O*-pentose, and *O*-deoxyhexose, respectively. Moreover, aglycones substituted with a linear glycoside molecule [peaks **9**, **14**] as well as disubstituted compounds were present in the **JM5** fraction [peak **15**]. **JM6** consisted of a mixture of flavonoid glycosides and was devoid of free aglycones. The LC–MS analysis is summarized in Table 1 and Supplementary Figures S1 and S2.

### 2.2. Identification of the Isolated Compounds **9**, **12** and **22**

**Figure 1:**
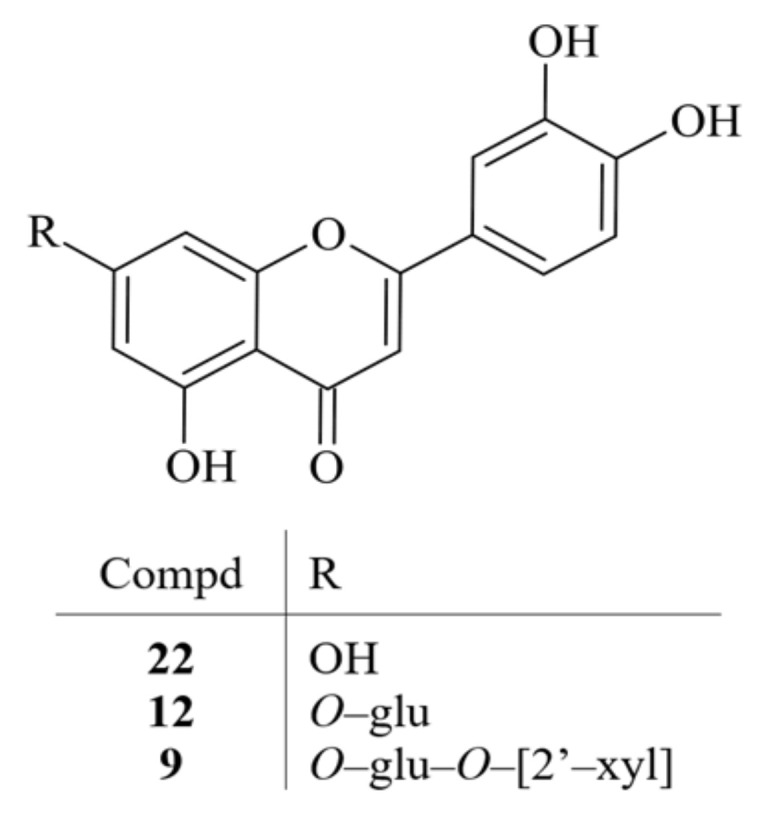
Chemical structures of isolated compounds **9**, **12** and **22**.

As a result of exhaustive multistep chromatographic isolation processes, three chromatographically homogeneous known compounds (**9**, **12**, and **22**) were isolated from the obtained **JM4**–**JM6** fractions. The identification of those compounds was carried out on the basis of R_f_ values, products of acid hydrolysis and spectroscopic methods (ultraviolet (UV) spectroscopy, nuclear magnetic resonance (^1^H NMR, ^13^C NMR), mass spectrometry (MS)). The spectral data of all compounds were identical to the available literature data. The spectral properties of compounds **9**, **12** and **22** were verified by comparison of its spectral data with those previously described in the literature. Isolated compounds were identified as luteolin 7-*O*-β-D-xylosyl-(1-2)-β-D-glucoside (luteolin 7-*O*-sambubioside, **9**), luteolin 7-*O*-β-D-glucoside (cynaroside, **12**) and luteolin (**22**) (Figure 1) [15,16,17] (Supplementary Figures S3–S8). 

### 2.3. Cytotoxicity Assay

**Figure 2:**
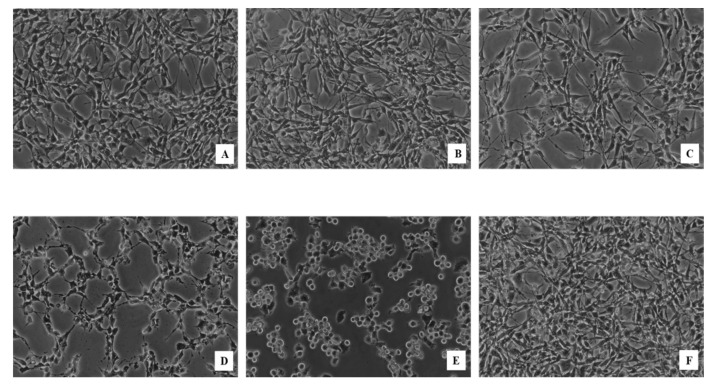
Morphological profile of C32 melanoma cells after 24 h of incubation with **JM4** in concentration 25 μg/mL (**A**), 50 μg/mL (**B**) and 100 μg/mL (**C**), **22** at concentration 25 μg/mL (**D**), and vinblastine sulfate (VLB) at concentration 25 μg/mL (**E**), compared with untreated control (**F**) evaluated by phase contrast microscopy (magnification × 200).

An MTT assay was performed separately for each sample for a preliminary assessment of the cytotoxic effects of **JM1**–**JM6** and compounds **9**, **12** and **22** on the viability of C32 and fibroblast cells. The MTT assay is among the most common methods for the evaluation of cell viability. However, several studies have shown inaccuracies that are inherent in the MTT method. Some studied plant extracts and polyphenols directly reduce the tetrazolium salt even in the absence of cells, which interferes with the MTT assay [18,19,20]. Additionally, the MTT assay has a limited operating range of compounds with a strong color that can be absorbed by the tested cells. Due to the imprecision of the MTT method, the fixable viability stain assay was chosen as an alternative to assess viability. C32 and fibroblast cells were treated with **JM1**–**JM6**, compounds **9**, **12**, **22** and vinblastine sulfate (VLB) at increasing concentrations (10–300 μg/mL) for 24 h. The results are presented in Table 2 and Supplementary Figures S9–S12. The morphological profile of C32 melanoma cells after 24 h of incubation with **JM4**, **22** and VLB is shown in Figure 2. We observed dose-dependent degradation of the cell membrane and a decrease in cell adhesion. Additionally, the number of cells in the treatment groups decreased compared with the untreated group, indicating inhibition of cell proliferation or induction of apoptosis. The highest activity was observed after treatment with compound **22** at a concentration of 25 μg/mL. The fixable viability stain study showed that C32 cell viability was inversely proportional to the applied concentration of all tested compounds. The cytotoxic potential is expressed as a median inhibitory concentration (IC_50_) value, where the IC_50_ value and cytotoxic activity value are inversely proportional. The tested extracts and compounds inhibited the viability of C32 cells in a dose-dependent manner. The IC_50_ values for some of the extracts and compounds were either similar (**JM4** IC_50_ = 119.7 μg/mL) to VLB (IC_50_ = 148.5 μg/mL) or somewhat higher (e.g., **JM6** IC_50_ = 215.7), while compound **22** had an IC_50_ value (95.1 μg/mL) even lower than that of VLB. The IC_50_ values of the other extracts were > 300 μg/mL. The viability of the fibroblast cells under the influence of **JM4** and **22** was higher than that of the C32 cells. In the case of fibroblasts, the IC_50_ value for **JM4** was above 300 μg/mL, and that for compound **22** was 194.1 ± 4.4 μg/mL (Supplementary Figure S13).

The obtained results suggested that **JM4** and compound **22** were the most active against human amelanotic melanoma C32 cells. Due to the high activity of the mentioned fraction and compound, **JM4** and **22** were submitted for further study. Notably, based on the content of compound **22** in **JM4**, it can be concluded that this compound is dominantly responsible for the observed effects of the **JM4** fraction on C32 cells.

### 2.4. Alteration of C32 Cell Cycle Progression by **JM4** and **22**

**Figure 3:**
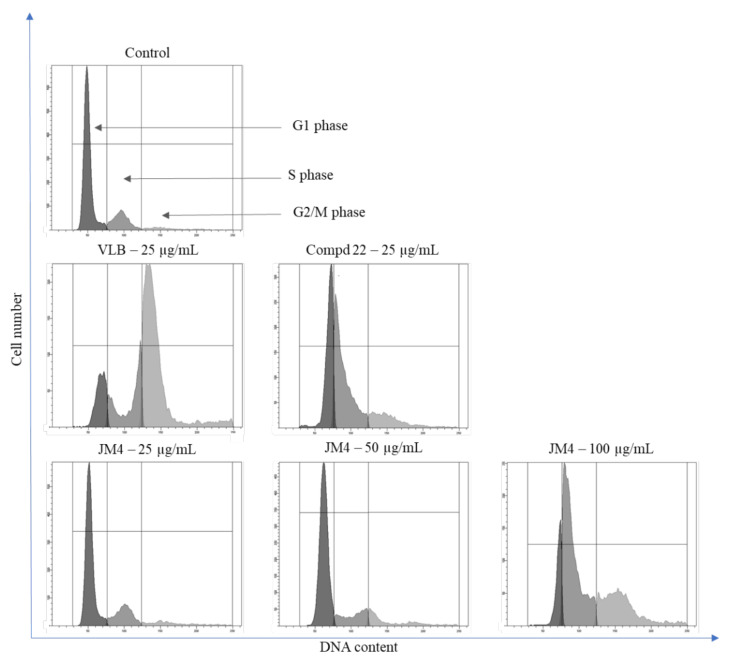
Flow cytometric analysis of cell cycle of C32 melanoma cells after 24 h of incubation with **JM4** (25, 50, 100 μg/mL), **22** (25 μg/mL) and vinblastine sulfate (VLB) (25 μg/mL) using propidium iodide (PI) staining.

**Figure 4:**
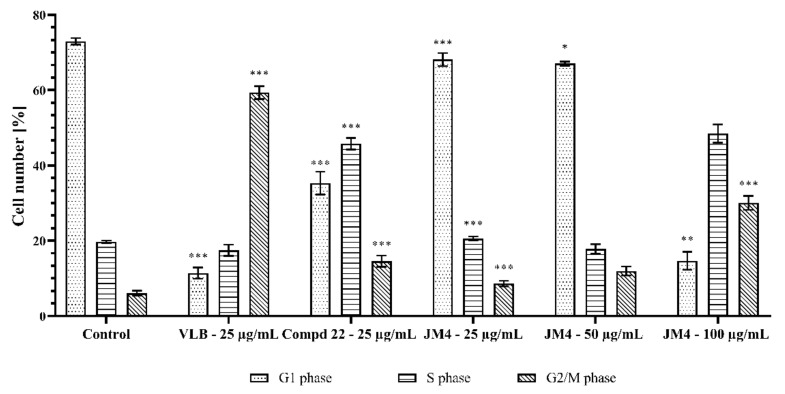
Percentage of accumulation C32 melanoma cells in the different cell cycle phases after 24 h of incubation with **JM4** (25, 50, 100 μg/mL), **22** (25 μg/mL) and vinblastine sulfate (VLB) (25 μg/mL). Mean percentage from three independent experiments (*n* = 3) done in duplicate are presented. * *p* < 0.05 versus control group, ** *p* < 0.01 versus control group, *** *p* < 0.001 versus control group.

The results demonstrated the differences between the investigated **JM4** and compound **22**, their concentration and the control group with respect to the percentage of accumulated C32 cells in different cell cycle phases after 24 h incubation (Figure 3 and Figure 4). **JM4** at a concentration of 100 μg/mL led to the accumulation of C32 cells in the S and G2/M phases. Simultaneously, the population of C32 cells in G1 phase after treatment with **JM4** was meaningfully reduced. The percentage of C32 cells in G2 phase increased from 6.1 ± 0.6% in the untreated control group to 30.1 ± 1.8% after treatment with **JM4** (100 μg/mL). However, this result was significantly lower than that in the case of VLB (25 μg/mL). Additionally, the percentage of C32 cells in G1 phase in the untreated control group decreased from 73.0 ± 0.9% to 35.3 ± 3.0% after incubation with **22** (25 μg/mL), and the percentage of C32 cells in G2/M phase increased from 6.1 ± 0.6% to 14.6 ± 1.5% after treatment with **22** (25 μg/mL).

The obtained results suggested that the effects on C32 cell cycle arrest in the S and G2/M phases was not caused by the main component of the **JM4** fraction, which is compound **22** (luteolin).

### 2.5. The **JM4** Fraction and Compound **22** Induce Apoptosis in Melanoma C32 Cells

During programmed cell death, annexin V has a high affinity for phosphatidylserine (PS) when it is exposed to the extracellular environment. Hence, this assay is based on the externalization of PS on the cell membrane. Additionally, propidium iodide (PI) was used to determine the number of necrotic cells, which confirms the integrity of the cell membrane [21,22]. The assay allows differentiability between viable cells (annexin V–/PI–) and early (annexin V–/PI+) and late (annexin V+/PI+) apoptotic cells, as well as necrotic cells (annexin V–/PI+).

**Figure 5:**
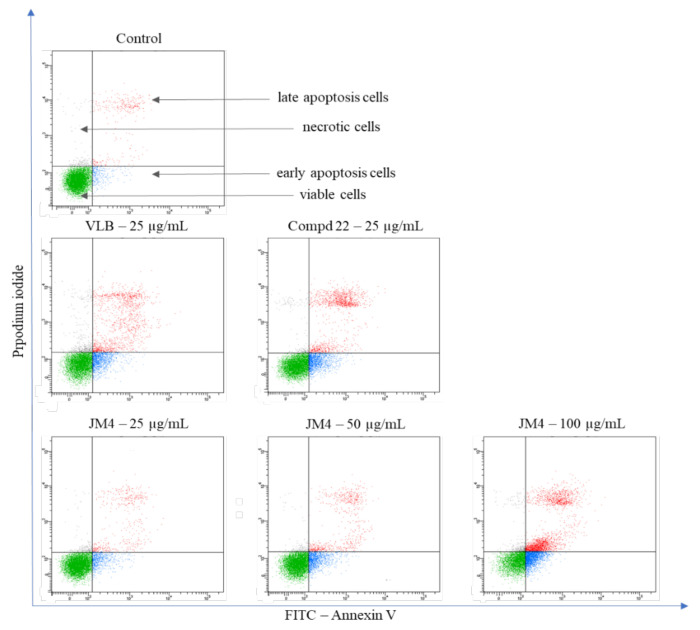
Flow cytometric analysis of C32 melanoma cells after incubation with **JM4** (25, 50, 100 μg/mL), **22** (25 μg/mL) and vinblastine sulfate (VLB) (25 μg/mL) for 24 h and subsequent staining with Annexin V and propidium iodide (PI).

**Figure 6:**
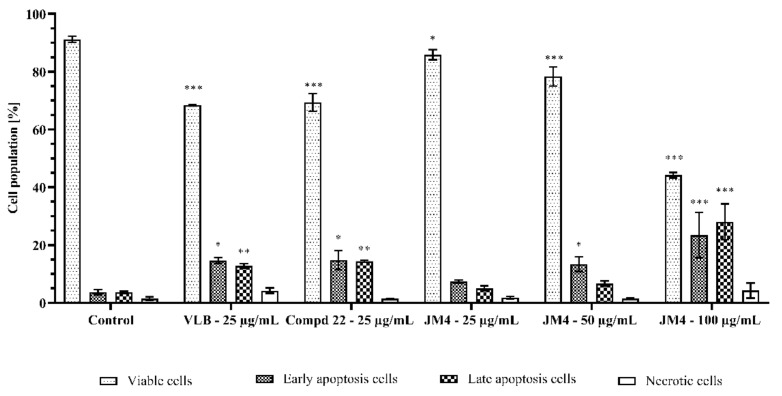
Percentage of viable C32 melanoma cells, early and late apoptosis cells, and necrotic cells, after 24 h of incubation with **JM4** (25, 50, 100 μg/mL), **22** (25 μg/mL) and vinblastine sulfate (VLB) (25 μg/mL). Mean percentages from three independent experiments (*n* = 3) done in duplicate are presented. * *p* < 0.05 versus control group, ** *p* < 0.01 versus control group, *** *p* < 0.001 versus control group.

An apoptosis study was performed to determine the mode of cell death provoked by **JM4** and **22**. The results are presented in Figure 5 and Figure 6. This analysis demonstrated that both tested samples, **JM4** and **22**, significantly induced programmed death in C32 cells in comparison with the untreated control group, where 91.2 ± 1.0% viable cells and 7.3 ± 1.3% apoptotic cells were observed. Fraction **JM4** (100 μg/mL) displayed the most significant proapoptotic effect after 24 h of incubation, where we detected 44.2 ± 1.0% viable cells and 51.5 ± 1.7% apoptotic cells. Nevertheless, with increasing concentration, a significant increase in the percentage of necrotic cells (4.3 ± 2.6% of necrotic cells after incubation with **JM4** (100 μg/mL)) was shown which may suggest the toxic activity of **JM4** at higher concentrations. Compound **22** (25 μg/mL) and VLB displayed similar proapoptotic effect after 24 h of incubation, where we detected 29.2 ± 3.0% apoptotic cells after treatment with **22**, and 27.4 ± 1.0% apoptotic cells after treatment with VLB. However, VLB exhibited two times higher necrotic potential (4.2 ± 1.0% necrotic cells after incubation with VLB (25 μg/mL)).

### 2.6. Fraction **JM4** and Compound **22** Induce Autophagy

Autophagy contributes to the maintenance of internal cell homeostasis by preventing the accumulation of damaged cell organelles. The level of autophagy is directly proportional to the increase in cellular stress, which ultimately leads to programmed cell death [23,24].

**Figure 7:**
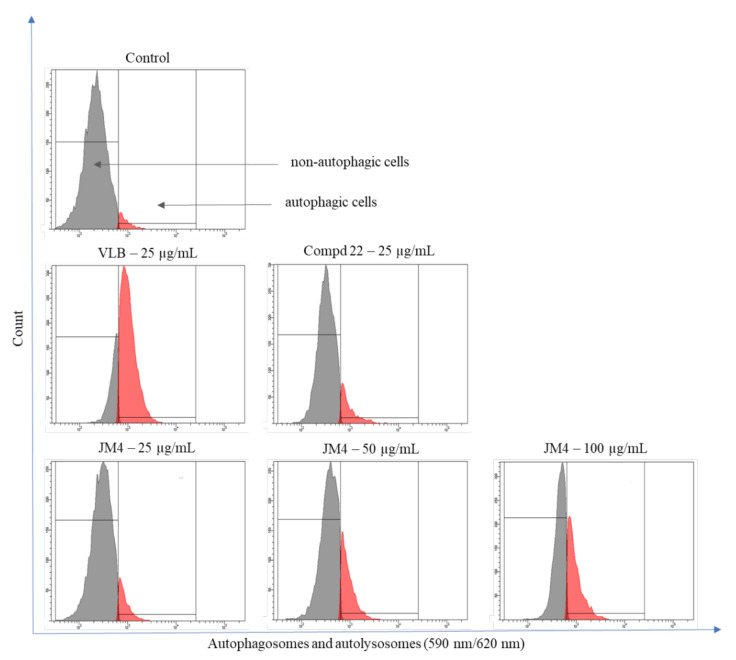
Autophagy induction in C32 melanoma cells measured by flow cytometry using Autophagy Probe (right—red histogram) compared to negative control cells (left—gray histogram) after 24 h incubation with **JM4** (25, 50, 100 μg/mL), **22** (25 μg/mL) and vinblastine sulfate (VLB) (25 μg/mL).

**Figure 8:**
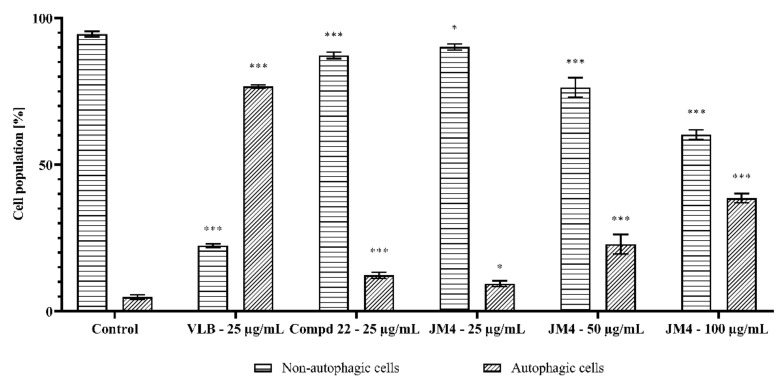
Percentage of nonautophagic and autophagic C32 melanoma cell population after 24 h incubation with **JM4** (25, 50, 100 μg/mL), **22** (25 μg/mL) and vinblastine sulfate (VLB) (25 μg/mL). Mean percentage values from three independent experiments (*n* = 3) done in duplicate are presented. * *p* < 0.05 versus control group, *** *p* < 0.001 versus control group.

The Autophagy Assay, Red was carried out via flow cytometry to detect the level of autophagy in C32 cells after 24 h of incubation with **JM4** and compound **22**. The results, expressed as the change in intensity of induced autophagy in C32 cells versus the untreated control group, differed according to treatment type (**JM4** vs. **22** vs. VLB) and concentrations (Figure 7 and Figure 8). There were 94.5 ± 1.0% nonautophagic cells and 4.8 ± 0.8% autophagic cells in the untreated control group. The highest dose-dependent activity of the induced autophagy process among the investigated samples/concentrations was displayed after 24 h incubation with fraction **JM4** (100 μg/mL), where we observed 60.2 ± 1.6% nonautophagic cells and 38.6 ± 1.6% of autophagic cells, which was twofold lower than in case of VLB. Significant activation of autophagy was observed after 24 h treatment with VLB (25 μg/mL); 22.4 ± 0.6% nonautophagic cells and 76.7 ± 0.5% of autophagic cells. The significant dose-dependent increase in the percentage of autophagic cells after **JM4** treatment may suggest that **JM4** causes stress in cells and leads to the induction of programmed cell death.

### 2.7. The Impact of Fraction **JM4** and Compound **22** on Mitochondrial Membrane Potential (MMP)

**Figure 9:**
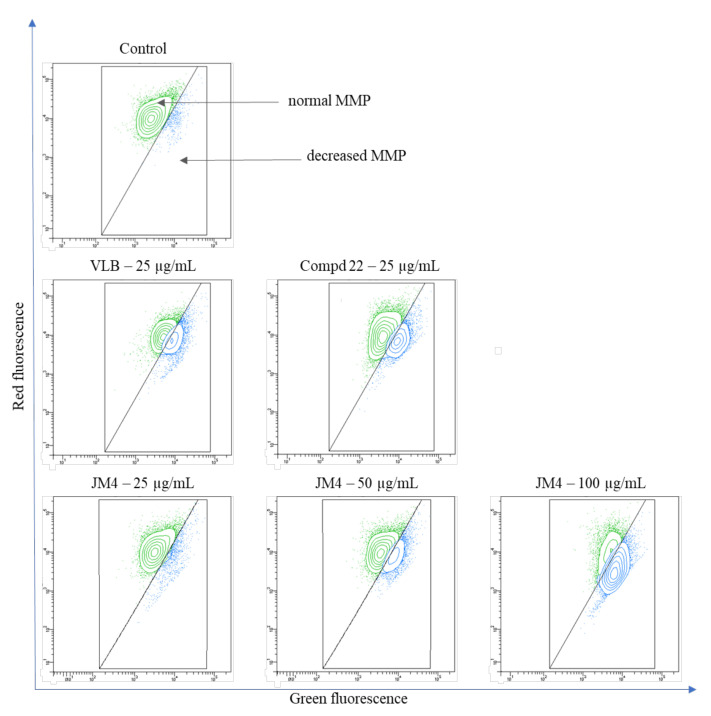
Fluorescence of C32 melanoma cells treated for 24 h with **JM4** (25, 50, 100 μg/mL), **22** (25 μg/mL) and vinblastine sulfate (VLB) (25 μg/mL) incubated with mitochondrial membrane potential probe JC-1.

**Figure 10:**
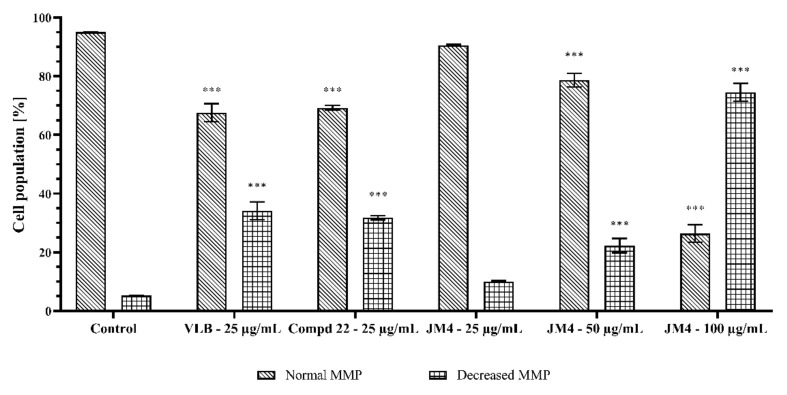
Percentage of C32 melanoma cells with normal and decreased mitochondrial membrane potential (MMP) after 24 h incubation with **JM4** (25, 50, 100 μg/mL), **22** (25 μg/mL) and vinblastine sulfate (VLB) (25 μg/mL). Mean percentage values from three independent experiments (*n* = 3) done in duplicate are presented. *** *p* < 0.001 versus control group.

To evaluate the mechanism underlying intrinsic cellular apoptosis under the influence of **JM4** and compound **22**, staining with the fluorescent dye JC-1 was performed. Reduced mitochondrial membrane potential (MMP) is associated with the early stages of apoptosis as well as with the appearance of cytochrome C in the cytosol [25,26,27]. As shown in Figure 9 and Figure 10, **JM4** caused a significant dose-dependent reduction in the MMP at the highest dose. We observed that 74.5 ± 3.0% of the cells had a reduced MMP after 24 h incubation with **JM4** (100 μg/mL), whereas the untreated control group had only a reduced MMP in 5.2 ± 0.1% of the cells. **JM4** at a concentration of 100 μg/mL led to a twofold higher percentage of cells with a reduced MMP than VLB (34.1 ± 3.0%). A weaker effect was displayed after 24 h of incubation with **22** (25 μg/mL), where we observed that 31.8 ± 0.7% of the cells had a reduced MMP. The obtained results are in accordance with the results from the annexin V binding assay.

### 2.8. Activation of Caspase-3, Caspase-8, Caspase-9, and Caspase-10

**Figure 11:**
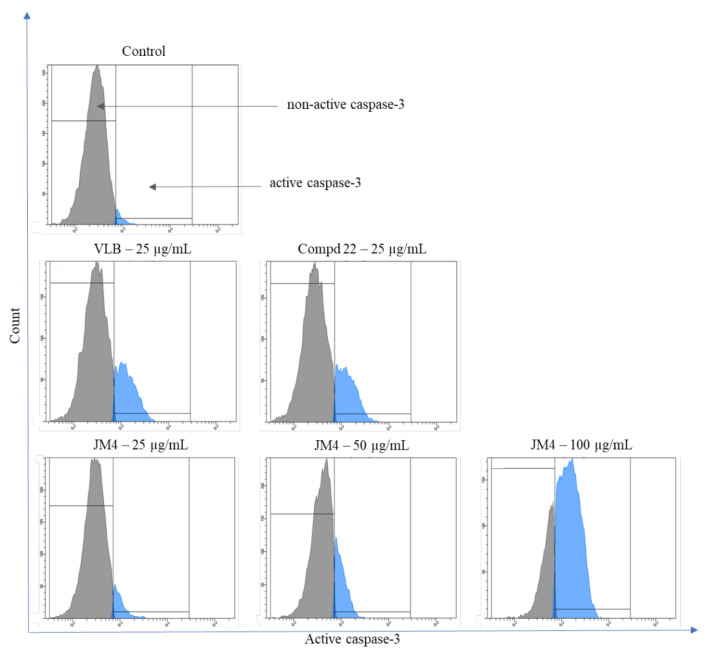
Flow cytometric analysis of populations C32 melanoma cells treated for 24 h with **JM4** (25, 50, 100 μg/mL), **22** (25 μg/mL) and vinblastine sulfate (VLB) (25 μg/mL) for active caspase-3.

**Figure 12:**
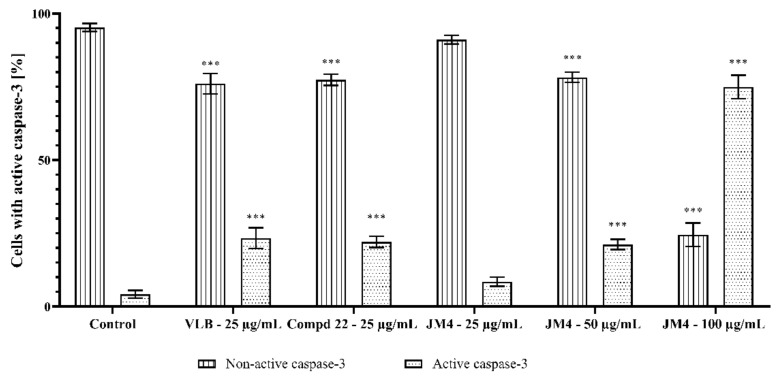
Percentage of C32 melanoma cells with nonactive and active caspase-3 after 24 h incubation with **JM4** (25, 50, 100 μg/mL), **22** (25 μg/mL) and vinblastine sulfate (VLB) (25 μg/mL). Mean percentage values from three independent experiments (*n* = 3) done in duplicate are presented. *** *p* < 0.001 versus control group.

**Figure 13:**
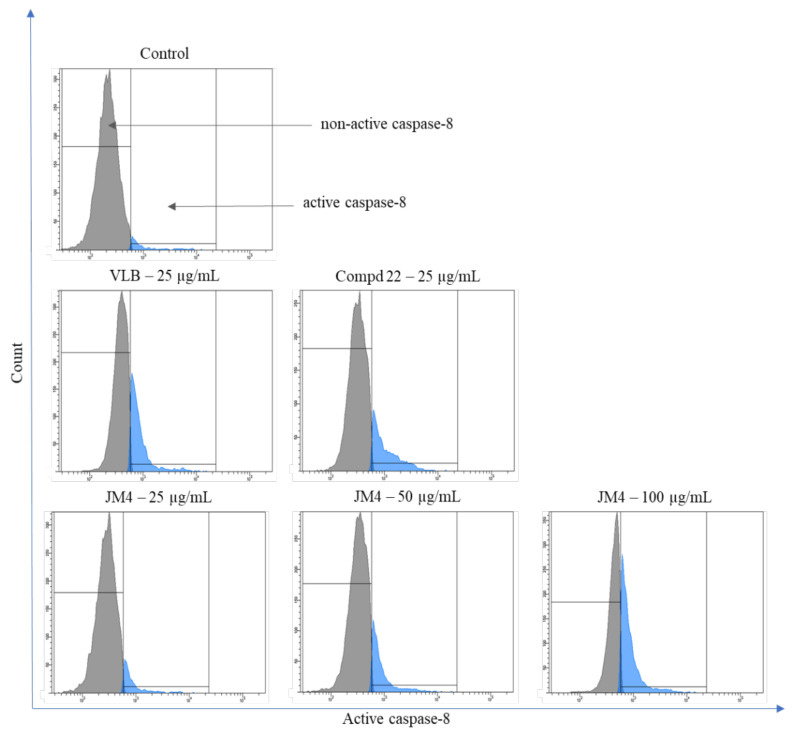
Flow cytometric analysis of populations C32 melanoma cells treated for 24 h with **JM4** (25, 50, 100 μg/mL), **22** (25 μg/mL) and vinblastine sulfate (VLB) (25 μg/mL) for active caspase-8.

**Figure 14:**
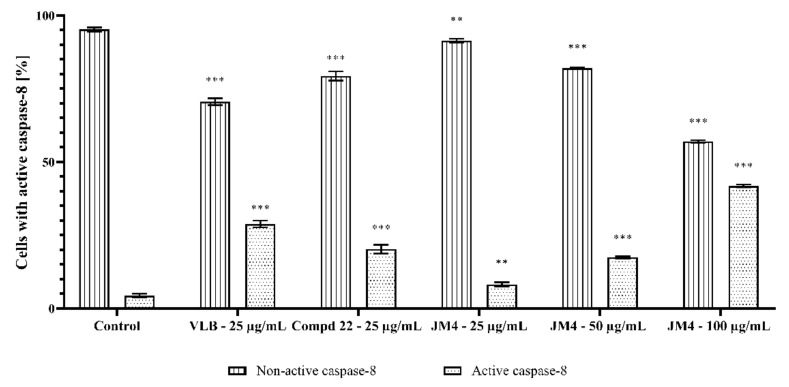
Percentage of C32 melanoma cells with nonactive and active caspase-8 after 24 h incubation with **JM4** (25, 50, 100 μg/mL), **22** (25 μg/mL) and vinblastine sulfate (VLB) (25 μg/mL). Mean percentage values from three independent experiments (*n* = 3) done in duplicate are presented. ** *p* < 0.01 versus control group, *** *p* < 0.001 versus control group.

**Figure 15:**
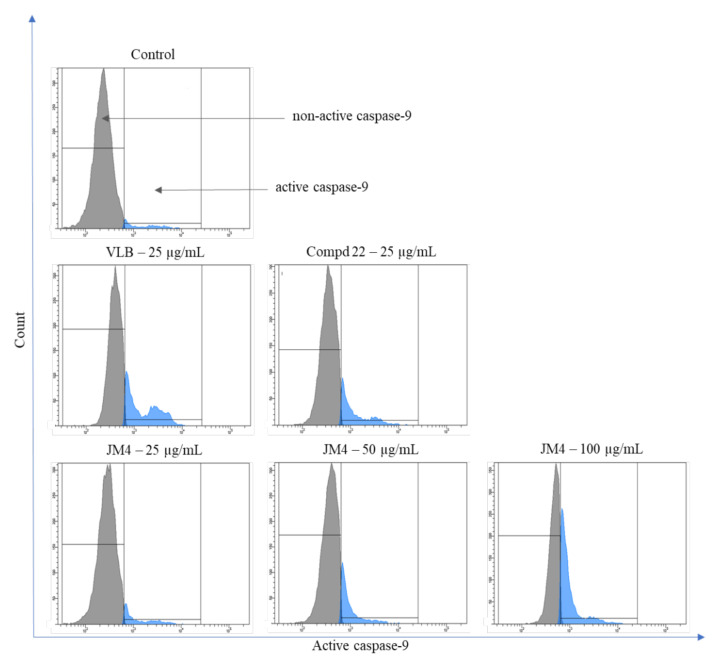
Flow cytometric analysis of populations C32 melanoma cells treated for 24 h with **JM4** (25, 50, 100 μg/mL), **22** (25 μg/mL) and vinblastine sulfate (VLB) (25 μg/mL) for active caspase-9.

**Figure 16:**
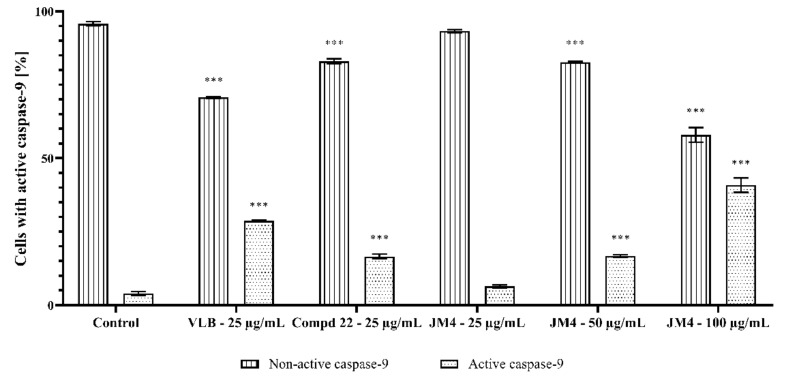
Percentage of C32 melanoma cells with nonactive and active caspase-9 after 24 h incubation with **JM4** (25, 50, 100 μg/mL), **22** (25 μg/mL) and vinblastine sulfate (VLB) (25 μg/mL). Mean percentage values from three independent experiments (*n* = 3) done in duplicate are presented. *** *p* < 0.001 versus control group.

**Figure 17:**
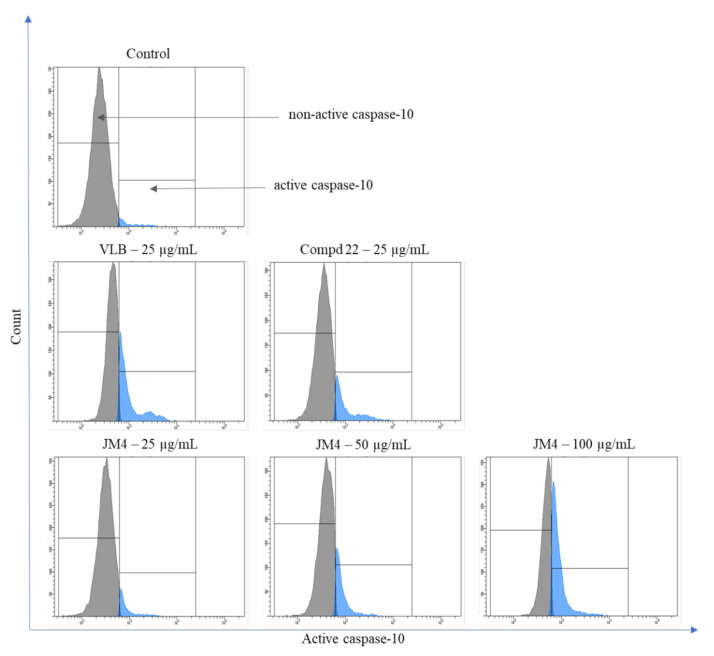
Flow cytometric analysis of populations C32 melanoma cells treated for 24 h with **JM4** (25, 50, 100 μg/mL), **22** (25 μg/mL) and vinblastine sulfate (VLB) (25 μg/mL) for active caspase-10.

**Figure 18:**
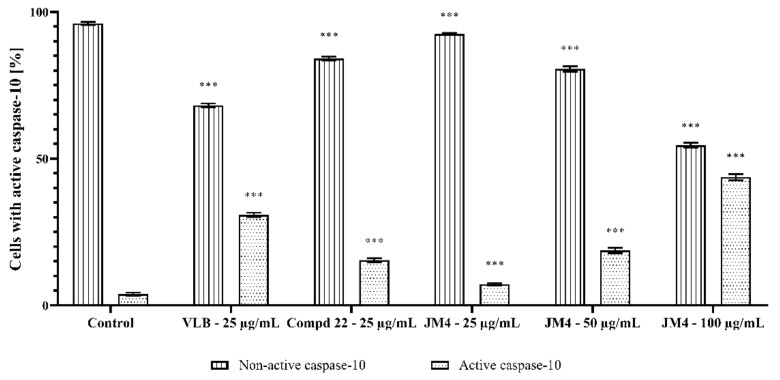
Percentage of C32 melanoma cells with nonactive and active caspase-10 after 24 h incubation with **JM4** (25, 50, 100 μg/mL), **22** (25 μg/mL) and vinblastine sulfate (VLB) (25 μg/mL). Mean percentage values from three independent experiments (*n* = 3) done in duplicate are presented. *** *p* < 0.001 versus control group.

Caspases play a crucial role in the activation of the apoptotic process. Activation of apical caspases (caspase-8 and caspase-10) leads to initiation of the external apoptotic pathway as well as activation of the effector caspase, caspase-3. In addition, the intrinsic (mitochondrial) apoptotic pathway is closely related to the activity of caspase-9, which is stimulated by a reduction in the MMP and plays an equally important role in the activation of caspase-3. Caspase-3 instructs the cell directly through apoptosis and DNA damage [28,29,30]. The influence of **JM4** and compound **22** on the expression of caspases 3, 8, 9, and 10 in C32 cells is shown in Figure 11, Figure 12, Figure 13, Figure 14, Figure 15, Figure 16, Figure 17 and Figure 18. The results proved that there was a significant increase in the levels of all caspases compared to the untreated control group after 24 h incubation with tested samples. The most significant effect of the activation of caspase-8, caspase-9, and caspase-10 was observed after incubation with **JM4** (100 μg/mL), where 41.8 ± 0.5% of cells had active caspase-8, 40.9 ± 2.4% of cells had active caspase-9, and 43.7 ± 1.1% of cells had active caspase-10. In all cases, the increases were almost twofold higher than after treatment with VLB. Compound **22** (25 μg/mL) displayed a slightly weaker impact on the activation of caspase-8, caspase-9, and caspase-10, with 20.2 ± 1.5%, 16.6 ± 0.8%, and 15.4 ± 0.7% of cells with the active respective caspase. According to these results, **JM4** and **22** induce the external and intrinsic apoptotic pathways as well as the activation of caspase-3, and **JM4** (100 μg/mL) is the strongest activator of caspase-3. After treatment with **JM4**, 74.9 ± 4.0% of the cells had active caspase-3, in comparison with 4.2 ± 1.3% of the cells in the untreated control group and 23.4 ± 3.5% of the cells after treatment with VLB. These results were consistent with the previous hypothesis of the intrinsic mitochondrial apoptotic pathway. The results showed that one of the molecular mechanisms leading to apoptosis caused by **JM4** is the activation of caspases in a dose-dependent manner. Our study confirmed that **JM4** was the strongest activator of all the tested caspases. Increasing the **JM4** concentration enhanced its cytotoxic properties and significantly increased the expression of caspase-3, which is responsible for DNA damage.

## 3. Discussion

The extracts and fractions obtained from the aerial parts of *J. montana* were used in this study. Due to the lack of literature data on the phytochemical profile and biological properties of *J. montana* as well as traditional anticancer drugs from *J. montana*, we performed the isolation and identification of the main compounds from the fractions from *J. montana* and examined their activity against human amelanotic melanoma C32 cells. Pathological skin cells were selected for this research due to the awareness of problems resulting from unknown plant matrix metabolic pathways when used internally. Additionally, previous studies have suggested the anticancer potential of luteolin and its derivatives [6,31,32,33]. To the best of our knowledge, there are currently no reports in the literature on the effects of *J. montana* extracts and its main phytoconstituents on C32 cells. 

The main phytochemicals belonging to the flavone group that were found and isolated from *J. montana* herbs include luteolin 7-*O*-sambubioside, luteolin 7-*O*-glucoside, and luteolin (**9**, **12**, and **22**, respectively). During the chromatographic study, it was found that the EtOAc fraction **JM4** contained luteolin (**22**) and luteolin 7-*O*-glucoside (**12**). **JM5** and the *n*-BuOH fraction **JM6** contained luteolin-7-*O*-sambubioside (**9**). These compounds were identified by comparison of the obtained UV spectral data as well as ^1^H and ^13^C NMR data with the available literature data, thus confirming their presence in the aerial parts of *J. montana*. The presence of these compounds in the studied species had already been described for the first time [12]. However, compound **9** was isolated for the first time from *J. montana*.

Luteolin displays numerous health-related properties, such as antiallergy, anti-inflammatory, antioxidant, anticancer activities and it was also reported as MAO-B inhibitors for the treatment of neurodegenerative diseases [34]. In traditional Chinese medicine, plants rich in luteolin have been used for the treatment of various disorders, such as inflammatory diseases, hypertension, and even malignant tumors [7,35]. Luteolin 7-*O*-glucoside (**12**) protects cells against apoptosis activated by hypoxia and buffers atopic dermatitis skin lesions in murine models [36].

The metabolic pathways of natural compounds and the complexity of the plant matrix have been insufficiently studied and pose a problem for internal administration. Hence, it seemed appropriate to undertake research towards only the potential external application of the raw material. Many anticancer compounds act by inhibiting tumor growth by arresting the cell cycle and/or inducing apoptosis [37]. The action of luteolin in the context of cancer, including melanoma, has been extensively described in review articles [8,31,32,33]. There are several suggested mechanisms by which luteolin may exert an inhibitory effect on human melanoma, including the induction of cell cycle arrest [38] or activation of apoptosis by increasing the level of intracellular reactive oxygen species (ROS) [39]. Additionally, the activity of luteolin was evaluated in an experimental in vivo metastasis model of hypoxia-induced epithelial–mesenchymal transition (EMT) inhibition through the regulation of β3 integrin [4]. This result was confirmed by luteolin research in melanoma cells, during which attempts were made to determine the influence of luteolin on the programming of the cell cycle, proving the arrest of this process in the G2/M and G0/G1 phases of the cell cycle [3]. Luteolin activates caspase-3, caspase-8, caspase-9, and caspase-10, activates DNA fragmentation factor, reduces MMP, and induces cytochrome C release into the cytosol [32]. Additionally, research on CH27 human lung squamous carcinoma cells showed that luteolin causes DNA damage and cell cycle arrest in S phase [40]. The aforementioned anticancer effects and mechanisms of luteolin are only examples of the very broad spectrum of action of this flavonoid [32].

The cytotoxic properties of chemotherapeutic substances are often associated with the induction of apoptosis in cancer cells [41]. Our results demonstrated the cytotoxic potential of the tested samples in amelanotic melanoma C32 cells. These melanoma cells showed a reduction in cell viability in a dose-dependent manner. The calculated IC_50_ value for all investigated extracts and fractions in the cytotoxic assays allowed us to observe differences in their activity. Luteolin (**22**) and fraction **JM4** showed the greatest cytotoxic potential that was comparable to that of VLB during the next steps of experiment. The presence of **22** in **JM4** was confirmed by LC–MS analysis, in which the presence of the ions 269 [M-H]^−^ and 271 [M-H]^+^ were found, which corresponded to the mass of the monoisotopic ion of luteolin, as well as the LC peak of the standard substance that had the same retention time as luteolin. It is worth noting that the compound **22** was the dominant in the analyzed fraction **JM4**. However, **JM4** was identified as a mixture of other polyphenolic compounds (Supplementary Figure S2).

Said and co-authors set out to determine the antiproliferative potential of flavonoids isolated from *Ailanthus excelsa*, including **12** and **22** against the same amelanotic melanoma C32 cell line used in this study as well as large cell lung carcinoma COR-L23 cells and malignant melanoma A375 cells. The results showed that luteolin had strong antiproliferative effects, comparable to those of VLB in all tested cell lines [42]. The observed antiproliferative effects of luteolin indicate that it might play a significant role in the overall activity of **JM4**.

While both **22** and **JM4** displayed notable degrees of cytotoxic activity in C32 cells in this study, their cytotoxic potential against normal fibroblast cells was much lower. To further elucidate the mechanism of cell damage and the possible induction of apoptosis, the ability of the test compounds to arrest the cell cycle was investigated. Flow cytometry analysis showed a decrease in the number of cells in G1 phase, which was most clearly seen in the case of **JM4**. Additionally, both **JM4** and compound **22**, at concentrations of 100 and 25 μg/mL, respectively, showed an increase in the accumulation of cells in the S and G2/M phases. Similar results were obtained in A375 melanoma cells. It has been shown that luteolin can contribute to cell cycle arrest and the induction of apoptosis by arresting the cell cycle at the G2/M and G0/G1 phases [3]. Literature data have confirmed that many antitumor substances block the cell cycle in G2/M phase [43,44,45]. It should be emphasized that there was a significant increase in the number of cells in S phase, which is a significantly higher result than in the case of VLB. The demonstrated disruptions in the cell cycle were associated with the inhibition of cell proliferation.

Apart from cell cycle arrest, mitochondria and caspases play an essential role in the induction of apoptosis. At the cellular level, many factors mediate apoptosis. One is the dysfunction of mitochondrial membrane integrity that can result in apoptotic cell death. Reducing the MMP causes the release of cytochrome c and other proteins. These proteins play an important role in the activation of caspase-9 and hence caspase-3, which directly leads to cell death [46,47,48]. To show the effects of the mitochondrial pathway on the apoptosis process, flow cytometry analysis was conducted and showed a significant decrease in MMP under the influence of **JM4** and compound **22**, thus confirming that apoptosis was triggered. Notably, the reduction in MMP in the case of compound **22** was similar to that caused by VLB, and in the case of **JM4**, it was twice as high as that recorded in the positive control.

The relationship between the reduction in MMP and the induction of the intrinsic apoptotic pathway is based on the activation of pro-caspase-3. In the context of cellular apoptosis, caspase-8 and caspase-10, which are involved in the external apoptotic pathway, are equally important [49]. As discussed in the results section, **JM4** and compound **22** exerted a significant antiproliferative effect in C32 cells that was associated with caspase-8, caspase-9, and caspase-10, followed by caspase-3. In a recent paper, Danciu et al. showed that chamomile flower extract presents a proapoptotic effect against human melanoma A375 cells. The dominant components of the studied extract were the following flavonoids: apigenin glucoside, rutin, luteolin glucoside, and luteolin. The chamomile flower extract activated caspase-3 at a concentration of 60 μg/mL. However, this flavone complex did not show significant activity in terms of cell cycle distribution or induction of apoptotic phases. This nonsignificant effect of the extract may be caused by the synergistic action of all components of the extract [50]. A similar effect was observed in study on a rosemary extract and human melanoma A375 cells where the extract was subjected to an MTT test with the main pure compounds such as rosmarinic acid, luteolin, carnosol, apigenin, and scutellarin. The dominant compound, rosmarinic acid, showed a much weaker antiproliferative effect than the extract. The substances whose contents in the extract were lower (carnosol, apigenin and luteolin) turned out to be more effective. However, since the individual substances were effective at concentrations well above those found in the rosemary extract, the results suggested that the cytotoxicity of the whole extract was due to the combination of activities of different substances [51].

Autophagy may have two functions: protumorigenic and antitumorigenic, depending on the type of cell, stage of cancer development and stimulator [24]. Most of the currently available chemotherapeutic agents exert their cytotoxic activity by promoting apoptosis. Previous reports have indicated that autophagy is essential in cancer therapy as well as apoptosis [52,53,54,55]. These assumptions are supported by studies on the effects of another natural polyphenol, curcumin, in melanoma A375 cells. Zhao et al. proved the relationship between the induction of cell proliferation and autophagy [56]. Our results led to similar conclusions by showing the activation of autophagy and apoptosis under the influence of **JM4** and compound **22**.

Thus, the induction of apoptosis in cancer cells treated with fraction **JM4** described in this study may be partly explained by the presence of luteolin (**22**). However, the conducted studies, which were aimed at demonstrating the induction of apoptosis in neoplastic cells under the influence of the tested fraction and pure compound, showed differences in the mitochondrial potential, caspase activation and the number of cells in the apoptotic phases between the samples used. This may be because the plant matrix of the fraction **JM4** is present and because of the possible synergism of the various phytochemical components such as compound **22** and its derivatives. Previous studies on luteolin (**22**) and its antitumor effects have confirmed its inhibitory effect against various types of cancer, including human lung carcinoma cells [40], oral squamous cancer cells [57], human esophageal adenocarcinoma cells [58], human colon cancer cells [59], human hepatoma cells [60], breast cancer, pancreatic cancer, prostate cancer, glioblastoma, and many others [7]. Hence, it is possible to hypothesize that *J. montana*, as a rich source of derivatives of luteolin, may be the basis for further research due to their potential antitumor activity. 

## 4. Materials and Methods

### 4.1. Chemicals and Reagents

Methanol (MeOH) (CAS67-56-1), petroleum spirit (CAS8032-32-4), chloroform (CHCl_3_) (CAS67-66-3), diethyl ether (Et_2_O) (CAS60-29-7), ethyl acetate (EtOAc) (CAS141-78-6), *n*-butanol (*n*-BuOH) (CAS71-36-3), ethanol (EtOH) (CAS64-17-5), Sephadex LH-20, vinblastine sulfate (VLB) (CAS143-67-9), dimethyl sulfoxide (DMSO) (CAS67-68-5), and 3-(4,5-dimethylthiazol-2-yl)-2,5-diphenyl tetrazolium bromide (MTT) (CAS298-93-1) obtained from Sigma Aldrich Co. (St. Louis, MO, USA). Polyamide (CAS25038-54-4), chrysoeriol and *p*-coumaric acid were purchased from Carl ROTH (Karlsruhe, Germany). The human amelanotic melanoma cell line C32 (CRL-1585) and a normal human fibroblast cell line (PCS-201-012) were purchased from the American Type Culture Collection (ATCC; Manassas, VA, USA). Dulbecco’s minimal essential medium (DMEM), fetal bovine serum (FBS), phosphate-buffered saline (PBS), glutamine, penicillin, and streptomycin were purchased from Corning (Corning, NY, USA). Propidium iodide (PI) (CAS 25535-16-4), stain buffer, and an FITC Annexin V Apoptosis Detection Kit II were obtained from BD Pharmingen (San Diego, CA, USA). Fixable Viability Stain 520 (FVS520) was from BD Horizon, San Diego, CA, USA and the DNase-free RNase A Solution was acquired from Promega, Madison, WI, USA. The Autophagy Assay, Red kit, FLICA Caspase-3/7 Assay Kit, FLICA Caspase-8 Assay Kit, FLICA Caspase-9 Assay Kit, and FLICA Caspase-10 Assay Kit were purchased from ImmunoChemistry Technologies (Bloomington, MN, USA). The JC-1 MitoScreen kit was from BD Biosciences Systems (San Jose, CA, USA). Ultra-pure water (UPW) for the preparation of the mobile phase for LC–PDA–ESI–MS/TOF analysis was performed on a POLWATER DL3-100 system (Labopol, Kraków, Poland). Acetonitrile (MeCN) (CAS75-05-08) was purchased from Fisher Chemical (Thermo Fisher Scientific, Leicestershire, UK), and both mobile phases were modified with the addition of formic acid (HCOOH) (CAS64-18-6) (Ph. Eur., Merck, Darmstadt, Germany). Apigenin (purity > 96%) and tricin (purity > 96%) were isolated from the inflorescences of *Arctium tomentosum* [61] and *Cirsium palustre* flower heads [62], respectively. Luteolin 7-*O*-sambubioside, luteolin 7-*O*-glucoside, and luteolin were isolated from *J. montana* as described below.

### 4.2. Plant Material

The aboveground parts of *J. montana* (2.0 kg) were collected from plants occurring in their natural habitat within the area of Puszcza Knyszyńska (N53°15′20.9″; E23°25′41.5″) in the region of Supraśl (Podlasie Province, Poland) within the period of June–August 2017 and 2018. The plant material was dried in a shaded and well-ventilated area. Samples of the collected plant material were identified based on the scientific botanical literature and its morphological features by one of the authors (M.T.). A plant voucher specimen (JM-15029) has been deposited in the Herbarium of the Department of Pharmacognosy at the Medical University of Białystok, Poland.

### 4.3. Preparation of Extracts **JM1**–**JM3** and Fractions **JM4**–**JM6**

The raw plant material (5 g per sample) was powdered and subsequently extracted using a heating mantle and radiator (5 × 45 min). All extractions were carried out using 65 mL of one of the following solvents: H_2_O (**JM1**), 50% MeOH (**JM2**), and MeOH (**JM3**). After filtration of the extracts, the solvents were evaporated under reduced pressure (BÜCHI System, Flawil, Switzerland) at a controlled temperature of 40 ± 2 °C. The remaining residues were suspended in water and lyophilized using a freeze-drier (Lyph-Lock, Labonco, Italy). The following amounts of the extracts were obtained: **JM1**, 1293 mg; **JM2**, 1238 mg; and **JM3**, 858 mg. Additionally, the plant material (130 g) was purified with a continuous extraction method using extraction petrol (1.5 L × 8 h), and then CHCl_3_ (1.5 L × 8 h) in Soxhlet extractor. The purified raw material was exhaustively extracted with MeOH (20 × 3 L) and 50% (*v/v*) MeOH (3 L) for 45 min each time. After the obtained MeOH extracts were combined and evaporated to dryness, they were precipitated with water for elimination of the ballasts. The obtained water extract was exhaustively fractionated by liquid–liquid extraction with different solvents of increasing polarity: CHCl_3_ (35 × 200 mL), Et_2_O (**JM4**; 50 × 200 mL), EtOAc (**JM5**; 98 × 200 mL) and *n*-BuOH (**JM6**; 45 × 200 mL). All fractions were evaporated to dryness and finally lyophilized using a freeze-drier. The three fractions were obtained in the following amounts: **JM4**, 8.5 g; **JM5**, 29 g; and **JM6**, 38.5 g and were used for further experiments. The chloroform fraction (CHCl_3_) was not investigated.

### 4.4. LC–ESI–MS Analysis of Extracts **JM1**–**JM3** and Fractions **JM4**–**JM6**

To establish the phytochemical compositions of **JM1**–**JM6**, LC–PDA–ESI–MS/TOF analysis was performed on a 1260 Infinity chromatography system hyphenated to a 6230 TOF mass spectrometer and Dual Agilent Jet Stream ESI (Agilent Technologies, Santa Clara, CA, USA). The MS conditions were as follows: electrospray ionization (ESI) source in both positive and negative ionization mode, a gas flow of 12 L/min, a gas temperature of 325 °C, a nebulizer pressure of 45 psi, and capillary voltages of 4500 and 2500 V for positive and negative ion modes, respectively. The analysis was performed using a Kinetex XB-C18 column (150 × 2.1 mm, 1.7 µm; Phenomenex, Torrance, CA, USA). The mobile phases were UPW (solvent A) and MeCN (solvent B), both with 0.1% HCOOH. The gradient started with the elution of 5% solvent B over 1.5 min. Then, within 22 min, the concentration of solvent B reached 28%, in 35 min it reached 75%, and finally in 45 min it reached 95%, with a linear gradient. After 3 min of maintaining the 95% B concentration, in 49 min, the system returned to its initial conditions, and conditioning continued for 6 min. The total run time of the analysis was 55 min at 25 °C. The injection volume was 1.0 µL, and the flow rate was 0.1 mL/min.

### 4.5. Identification and Isolation of Main Compounds **9**, **12** and **22** (**JM7**–**JM9**)

Fractions **JM4**–**JM6** underwent labor-intensive isolation procedures with low pressure liquid chromatography (LPLC) to isolate the active compounds using various adsorbents, such as polyamide and Sephadex LH-2. The fractionation process was controlled by thin layer chromatography (TLC) (TLC Cellulose, 20 × 20 cm, MERCK, *n*-BuOH:CH_3_COOH:H_2_O, 4:1:5 (*v*/*v*/*v*); upper layer, sprayed with 1% Naturstoff reagent A) analysis under UV light. Isolated, chromatographically homogeneous compounds **9** (250 mg), **12** (1640 mg), and **22** (139 mg) were subjected to spectral analyses to determine their full structural characteristics. Spectral measurements were performed using UV-VIS (SPECORD 200 Plus, Analytik Jena, Jena, Germany) with various complexing reagents and ^1^H NMR and ^13^C NMR spectra were recorded (BRUKER Advance II 400, BRUKER, Billerica, MA, USA). The final characteristics of all isolated compounds were also confirmed by MS (Agilent, Santa Clara, CA, USA). 

### 4.6. Biological Assays

#### 4.6.1. Cell Culture

The human amelanotic melanoma cell line C32 (CRL-1585) and a normal human fibroblast cell line (PCS-201-012) were cultured in DMEM. DMEM was blended with 10% FBS, 10 µg/mL streptomycin, and 10 units/mol penicillin. The cells were cultured in 5% CO_2_ and fully humidified at 37 °C. All tested compounds were used at a final DMSO concentration of not more than 0.5% (*v/v*). Cells cultured in drug-free DMEM were used as controls, and cells with the addition of only DMSO were used as solvent-controls; VLB was used as a positive control. The **JM4** fraction was analyzed at the following concentrations: 25, 50 and 100 µg/mL and compound **22** and VLB were analyzed at 25 µg/mL. For the cytotoxicity assay, all extracts and compounds were tested at the following concentrations: 10, 25, 50, 100, 200, and 300 µg/mL.

#### 4.6.2. Cytotoxicity Assay

Cytotoxicity was evaluated by the MTT colorimetric assay previously described by Carmichael [63]. The MTT test is founded on the reduction of a yellow tetrazolium salt to purple formazan crystals by viable cells. C32 and fibroblast cells were seeded in 24-well plates at an initial density of 1 × 105 cells per well. The cultured cells were grown at 37 °C for 24 h and incubated with **JM1**–**JM6** and compounds **9**, **12** and **22** at various concentrations for 24 h. Each sample was dissolved in DMSO (the DMSO concentration was no greater than 0.5%) and further diluted in serum-free DMEM to achieve different concentrations (10–300 µg/mL). After incubation, 10 µL of MTT solution (5 mg/mL) was added to all cultured cells and followed by an additional incubation for 4 h (fibroblast cell line) or 10 min (C32 cell line). Upon removal of the medium, 200 µL of DMSO was added to all wells to dissolve the insoluble formazan. The results were measured spectrophotometrically at 570 nm using an Evolution 201 reader (Thermo Scientific, Waltham, MA, USA).

#### 4.6.3. Fixable Viability Stain Assay

This analysis was performed to estimate the level of viable cells after treatment with **JM1**–**JM6**, compounds **9**, **12**, **22** and VLB at concentrations ranging from 10 to 300 µg/mL. Cytotoxicity was evaluated by FVS520 via a flow cytometer (BD FACSCanto II flow cytometer, San Jose, CA, USA). In contrast to nonpermeable live cells, the permeable membranes of necrotic cells allow for the intracellular diffusion of FVS520 and the covalent binding of high concentrations of amines. Thus, necrotic cells contained a higher level of FVS520 and, consequently, increased fluorescence intensity than viable cells. After 24 h of incubation, C32 cells and fibroblasts were washed with PBS, trypsinized and resuspended in DMEM. The supernatant was removed, and 0.5 mL of Stain Buffer with the addition of 1 µL of FVS520 was added to each sample, which was vortexed immediately and kept for 10–15 min in the dark at room temperature. After incubation and the addition of 2 mL of PBS, the supernatant was removed, the cells were resuspended in 300 µL of PBS and then immediately subjected to analysis. The results were analyzed based on FACSDiva software (BD Biosciences Systems, San Jose, CA, USA). Based on the results of the cytotoxicity analysis, further research in this project focused on the determination of the full anticancer mechanism of the most promising fraction **JM4** and compound **22**.

#### 4.6.4. Cell Cycle Analysis

The allocation of cell cycle stage was analyzed on a FACSCanto II flow cytometer. C32 cells were cured with **JM4**, **22** and VLB for 24 h of incubation. Thereafter, the cells were harvested and adjusted with 1 mL of 70% EtOH and maintained at −20 °C for 24 h. Next, the cells were washed with PBS, treated with 50 µg/mL DNase-free RNase A solution for 5 min at room temperature, stained with 100 µg/mL PI for 30 min at 37 °C, and submitted to flow cytometry analysis. The results were analyzed with FACSDiva software.

#### 4.6.5. Flow Cytometry Assessment of Annexin V Binding

Characterization of the mode of apoptosis induced by **JM4**, **22** and VLB was performed in C32 cells via flow cytometry using a FITC Annexin V Apoptosis Detection Kit II. All stages of programmed cell death could be identified by annexin V bound with high affinity to PS. PI is a standard flow cytometric viability explorer that stains cells that interfere with the cell membrane, and it can be used to distinguish necrotic cells from dead cells. After the C32 cells were incubated for 24 h with various concentrations of **JM4**, **22** and VLB, they were trypsinized and resuspended in binding buffer. After that, 5 µL of annexin V-FITC and 5 µL of PI were added and maintained for 15 min in the dark at room temperature. The results were analyzed using FACSDiva software.

#### 4.6.6. Determination of the Level of Autophagy by Autophagy Assay, Red

An autophagy assay was carried out to detect the impact of **JM4**, compound **22**, and VLB on the autophagy process of C32 cells via flow cytometry using an Autophagy Assay, Red kit. The probe is a cell-permeable aliphatic molecule that brightly fluoresces after unjumbling autophosphates and autolysosomes into lipid membranes. Samples of C32 cells incubated for 24 h with **JM4**, **22**, and VLB were subjected to a rising procedure with PBS and then resuspended in PBS with the accessory of Autophagy Probe, Red solution, and maintained for 30 min at 37 °C in the dark. After incubation, the cells were flushed again and resuspended in cellular assay buffer. The results were analyzed with FACSDiva software.

#### 4.6.7. Analysis of Mitochondrial Membrane Potential (MMP)

Disorder of the MMP was determined by the lipophilic cationic probe 5,5’,6,6’-tetrachloro-1,1’,3,3’-tetrarthylbenzimidazol-carbocyanine iodide using a JC-1 MitoScreen kit. Samples of C32 cells after the 24 h incubation with **JM4**, compound **22**, and VLB, were subjected to a rising procedure with PBS, suspended in PBS with 10 µg/mL JC-1 and maintained for 15 min at room temperature in the dark. After incubation, the cells were flushed again, resuspended in PBS, and immediately subjected to BD FACSCanto II flow cytometry analysis. The results were analyzed using FACSDiva.

#### 4.6.8. Determination of Caspase-3, Caspase-8, Caspase-9, and Caspase-10 Activity

Determination of the caspase-3, caspase-8, caspase-9, and caspase-10 activity were carried out by the following kits: FLICA Caspase-3/7 Assay Kit, FLICA Caspase-8 Assay Kit, FLICA Caspase-9 Assay Kit, and FLICA Caspase-10 Assay Kit using a BD FACSCanto II flow cytometer. C32 cells were incubated with **JM4**, compound **22** and VLB for 24 h, washed twice with PBS, resuspended in buffer supplemented with 5 mL of FLICA reagent, and incubated. After 1 h, the cells were flushed with apoptosis wash buffer and resuspended in 100 mL of the same reagent with 10 mg/mL PI. The procedure of determining the activity of each caspase was carried out analogously. The results were analyzed with FACSDiva software.

#### 4.6.9. Cell Morphological Analysis

Visualization of the nuclear morphology of C32 cells was evaluated using a phase contrast microscope (Nikon Eclipse Ti, Tokyo, Japan) at 200× magnification. C32 cells (2.5 × 10^5^) were incubated with **JM4** at concentrations of 25, 50, and 100 µg/mL and compound **22** and VLB at a concentration of 25 µg/mL in 6-well plates for 24 h at 37 °C. After incubation, the cells were washed twice with PBS and observed under a phase contrast microscope.

#### 4.6.10. Statistical Analysis

All numerical data are shown as the mean ± standard deviation (SD) from at least three independent repeats. Statistical analysis was performed using GraphPad Prisma 8 software (GraphPad Software, San Diego, CA, USA). Statistical differences were assessed using one-way ANOVA followed by Tukey’s test. Values of *p* < 0.05 were considered statistically significant.

## 5. Conclusions

Summarizing, the results of conducted studies indicate that *J. montana* is a rich source of polyphenolic compounds, mainly luteolin and its derivatives demonstrated significant cytotoxic and proapoptotic potential. The obtained results have shown that *J. montana* may be an effective strategy to develop preparation with potential antimelanoma properties. However, further studies are required in the human system to determine cellular uptake, distribution, and the long-term effect of the isolated flavonoids in the skin.

## Figures and Tables

**Table 1:** Liquid chromatography–mass spectrometry (LC–MS) analysis of extracts/fractions (**JM1**–**JM6**) from *Jasione montana*.

Peak/Compound	Rt (min)	UV-VIS Maxima (nm)	[M–H]^−^ Ions (*m/z*)	[M–H]^+^ Ions (*m/z*)	Identified Compounds
**1**	4.60	260	191, 217, **235**	86, 136, **276**	Unknown
**2**	14.53	260, 294	**109**, 153, 277	93, **137**, 213, 248	Unknown
**3**	19.29	256, 310 sh	**93**, 183	**94**, 302	*p*-coumaroyl acid derivatives
**4**	20.79	268, 330	565, **771**	302, 538, **773**	Unknown
**5**	21.61	272, 330	593	415, **432**, 595	Flavonoid derivatives
**6**	22.53	268, 336	609	287, 449, **611**	Luteolin *O*-hex-hex
**7**	23.40	268, 338	563, **741**	287, 449, 565, **743**	Luteolin *O*-hex-pent-hex
**8**	24.41	268, 340	609	287, 449, **611**	Luteolin *O*-hex-hex
**9**	25.59	268, 348	285, **579**	287, 449, **581**	Luteolin 7-*O*-sambubioside (s)
**10**	25.87	310	**119**, 162	91, 119, **147**, 165	*p*-coumaric acid (s)
**11**	26.11	268, 338	593	271, 433, **595**	Apigenin *O*-hex-hex
**12**	26.84	258, 266, 348	**447**, 895	287, **449**	Luteolin 7-*O*-glucoside (s)
**13**	26.99	268, 326	593	287, 449, **595**	Luteolin *O*-hex-deoxyhex
**14**	27.46	250, 268, 336	285, 447, 609, **755**	287, 449, 611, **757**	Luteolin *O*-hex-hex-deoxyhex
**15**	27.85	268, 330	269, **563**	271, **418**, 565	Apigenin *O*-deoxyhex-*O*-deoxyhex
**16**	28.76	268, 335	285, 447, **579**, 769	287, 449, 581, **771**	Luteolin *O*-hex-pent-feruloyl
**17**	29.27	268, 330	285, **431**	286, **433**	Flavonoid derivatives
**18**	29.45	268, 336	285, **447**	287, **449**	Luteolin *O*-hex
**19**	30.17	268, 284	431	301, **419**, 571	Flavonoid derivatives
**20**	30.53	268, 300 sh, 340	285, **447**	287, **449**	Luteolin *O*-hex
**21**	32.36	270, 324	299, **461**	331, **463**	Tricin *O*-pent
**22**	34.38	256, 266, 348	285	287	Luteolin (s)
**23**	36.23	268, 295 sh, 340	269	271	Apigenin (s)
**24**	36.35	270, 300 sh, 349	329	331	Tricin (s)
**25**	36.54	268, 300 sh, 344	299	301	Chrysoeriol (s)

sh—peak shoulder; bold—most abundantion; s—reference substance; hex—hexose, pent—pentose, deoxyhex—deoxyhexose.

**Table 2:** IC_50_ of viability of CRL-1585 human amelanotic melanoma cells treated for 24 h with different concentrations of **JM1**–**JM6** and isolated compounds **9**, **12**, **22** from *J. montana*. Mean values ± SD from three independent experiments done in duplicate are presented. (IC_50_ value in μg/mL).

Sample	IC_50_ [μg/mL]
**JM1**	>300
**JM2**	>300
**JM3**	>300
**JM4**	119.7 ± 3.2
**JM5**	>300
**JM6**	215.7 ± 21.2
Compound **9**	>300
Compound **12**	>300
Compound **22**	95.1 ± 7.2
VLB	148.5 ± 7.7

## Supplementary Materials

The following are available online at https://www.mdpi.com/1422-0067/22/7/3345/s1, Figure S1: The qualitative assessment of J. montana extracts (JM1–JM3). UV-VIS chromatogram (λ = 280 nm) obtained by LC–PDA–MS, Figure S2: The qualitative assessment of J. montana fractions (JM4–JM6). UV-VIS chromatogram (λ = 280 nm) obtained by LC–PDA–MS, Figure S3: 1H NMR spectrum (400.15 MHz, DMSO-d6) of compound 9, Figure S4: 13C NMR spectrum (400.15 MHz, DMSO-d6) of compound 9, Figure S5: The MS spectrum of compound 9 in positive ion mode, Figure S6: 1H NMR spectrum (400.15 MHz, DMSO-d6) of compound 12, Figure S7: 13C NMR spectrum (400.15 MHz, DMSO-d6) of compound 12, Figure S8: 1H NMR spectrum (400.3 MHz, DMSO-d6) of compound 22, Figure S9: Flow cytometric analysis of cytotoxicity of C32 melanoma cells after 24 h of incubation with DMSO and VLB (10, 25, 50, 100, 200, and 300 μg/mL) comparable with untreated control by the fixable viability stain assay, Figure S10: Flow cytometric analysis of cytotoxicity of C32 melanoma cells after 24 h of incubation with JM1–JM3 (10, 25, 50, 100, 200, and 300 μg/mL) comparable with untreated control by the fixable viability stain assay, Figure S11: Flow cytometric analysis of cytotoxicity of C32 melanoma cells after 24 h of incubation with JM4–JM6 (10, 25, 50, 100, 200, and 300 μg/mL) comparable with untreated control by the fixable viability stain assay, Figure S12: Flow cytometric analysis of cytotoxicity of C32 melanoma cells after 24 h of incubation with compound 9, 12, and 22 (10, 25, 50, 100, 200, and 300 μg/mL) comparable with untreated control by the fixable viability stain assay, Figure S13: Flow cytometric analysis of cytotoxicity of normal human fibroblasts cells after 24 h of incubation with DMSO, JM4, and compound 22 (10, 25, 50, 100, 200, and 300 μg/mL) comparable with untreated control by the fixable viability stain assay.

## Abbreviations

CHCl_3_	chloroform
CMM	cutaneous malignant melanoma
DMEM	Dulbecco’s minimal essential medium
DMSO	dimethyl sulfoxide
ESI	electrospray ionization
Et_2_O	diethyl ether
EtOAc	ethyl acetate
EtOH	ethanol
FBS	fetal bovine serum
HCOOH	formic acid
H_2_O	water
IC_50_	median inhibitory concentration
LPLC	low pressure liquid chromatography
LC–PDA–ESI–MS/TOF	liquid chromatography–photodiode array detection–electrospray ionization–mass spectrometry
MeCN	acetonitrile
MeOH	methanol
MMP	mitochondrial membrane potential
MS	mass spectrometer
NMR	nuclear magnetic resonance
MTT	3-(4,5-dimethylthiazol-2-yl)-2,5-diphenyl tetrazolium bromide
*n*-BuOH	*n*-butanol
PBS	phosphate-buffered saline
PDA	photodiode array detection
PI	propidium iodide
PS	phosphatidylserine
ROS	reactive oxygen species
TLC	thin layer chromatography
UPW	ultra-pure water
UV	ultraviolet
UV-VIS	ultraviolet-visible spectroscopy
WHO	World Health Organization
VLB	vinblastine sulfate
