# Supplementary material for: In Vitro Anticancer Potential of Jasione montana and Its Main Components against Human Amelanotic Melanoma Cells
Source: Int J Mol Sci. 2021 Mar 25;22(7):3345. doi: 10.3390/ijms22073345 (PMC8036727; doi:10.3390/ijms22073345)
Supplement: Supplementary file 1 [file ijms-22-03345-s001.pdf]

International Journal of
*Molecular Sciences*

**SUPPLEMENTARY MATERIAL**

***In vitro* anticancer potential of *Jasione montana* and its main components against human amelanotic melanoma cells**

Aleksandra Maria Juszczak, Robert Czarnomysy, Jakub Władysław Strawa, Marijana Zovko-Končić, Krzysztof Bielawski and Michał Tomczyk

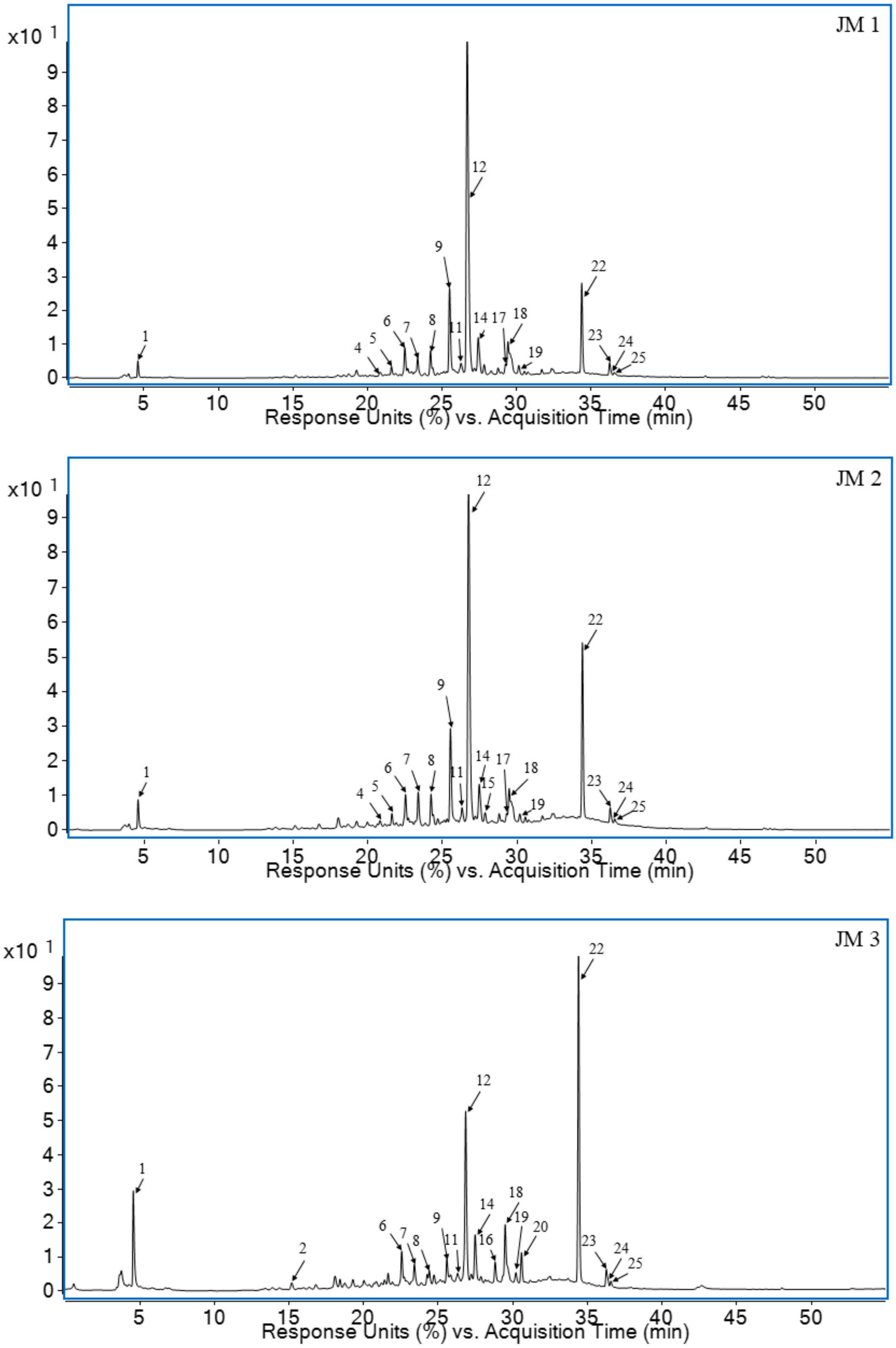


**Figure S1.** The qualitative assessment of *J. montana* extracts (**JM1–JM3**). UV-VIS chromatogram (λ=280 nm) obtained by LC-PDA–MS.

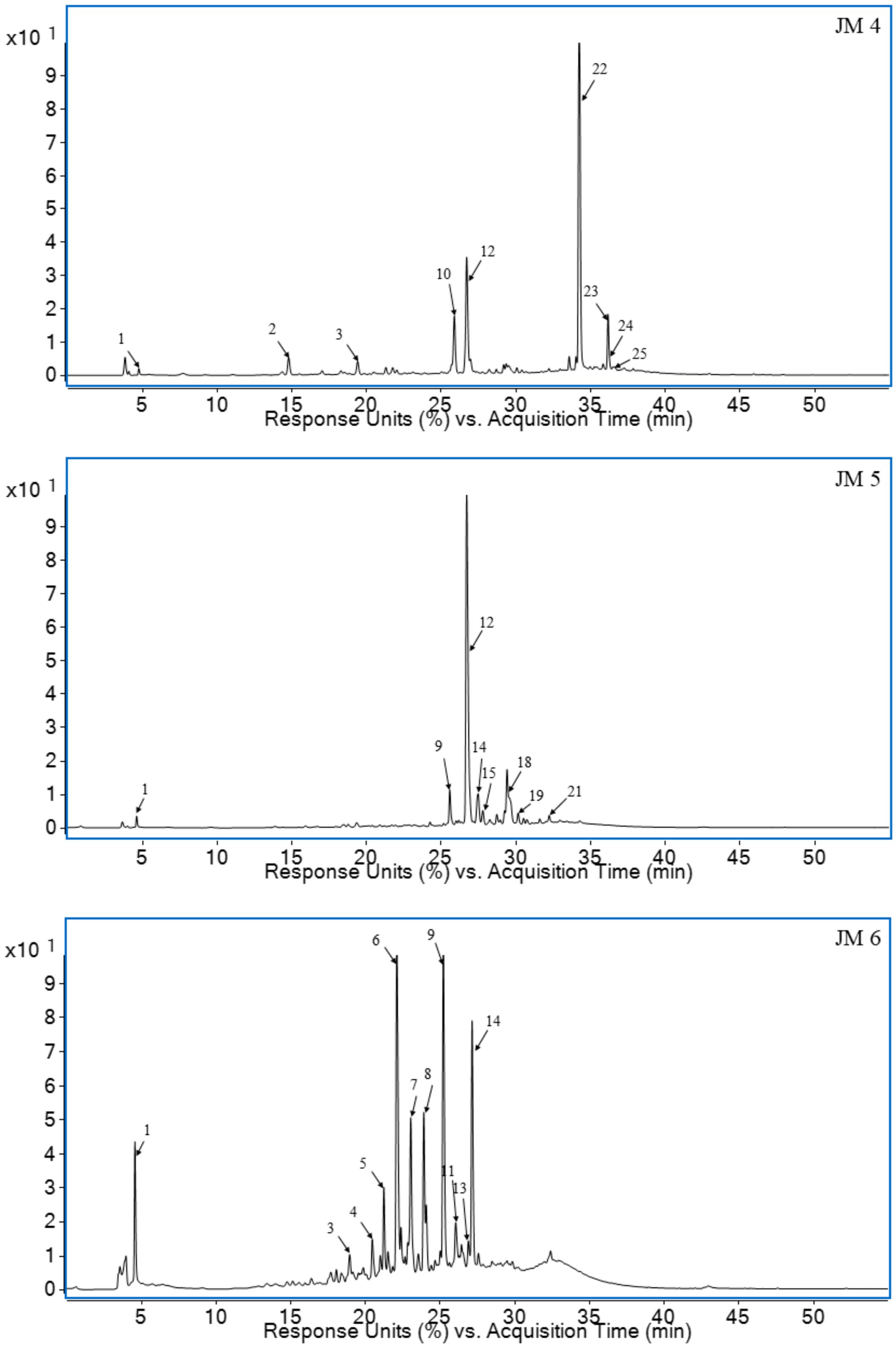


**Figure S2.** The qualitative assessment of *J. montana* fractions (**JM4–JM6**). UV-VIS chromatogram (λ=280 nm) obtained by LC-PDA–MS.

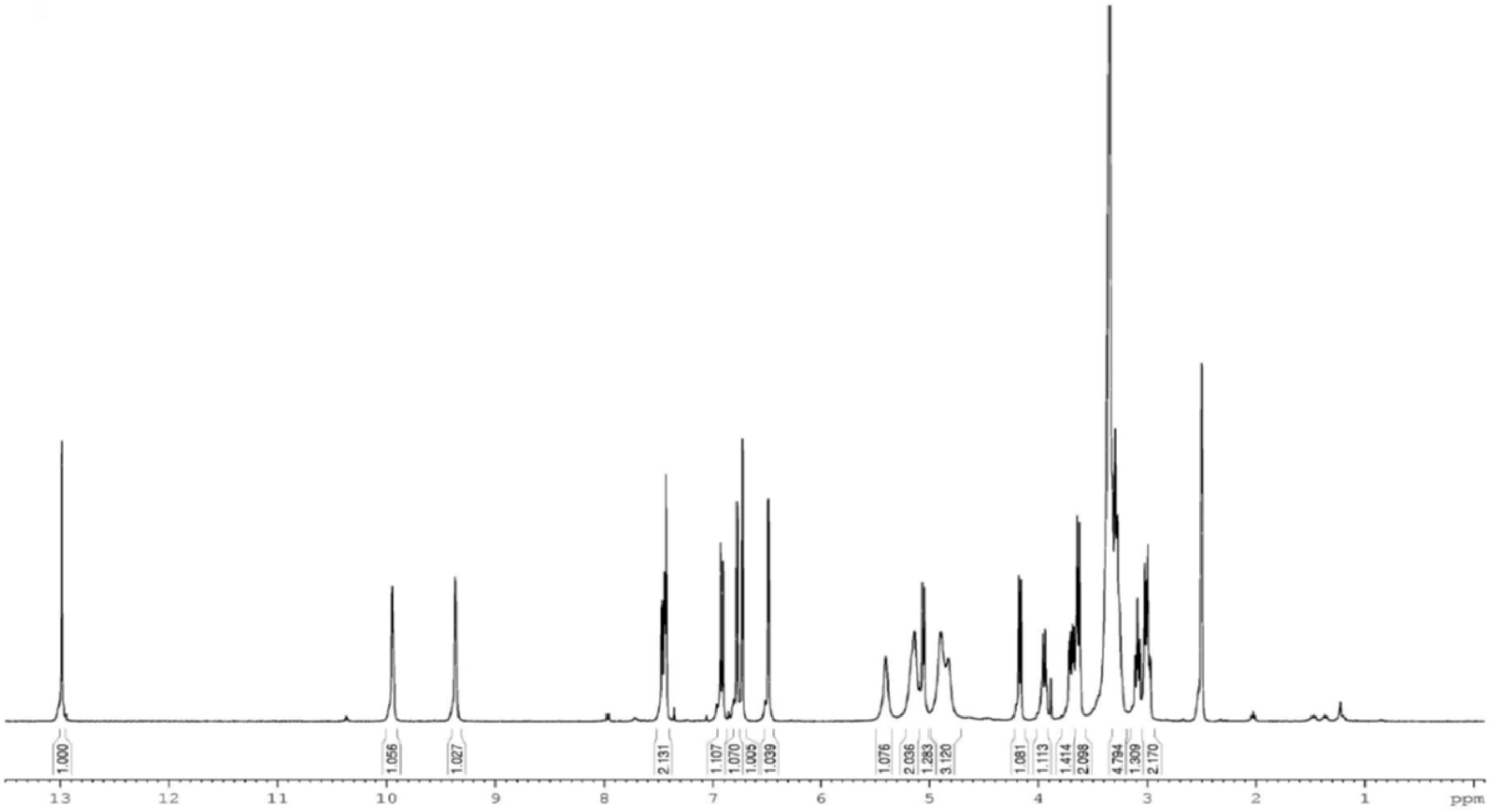


**Figure S3.** $^{1}$H NMR spectrum (400.15 MHz, DMSO-$d_6$) of compound **9**.

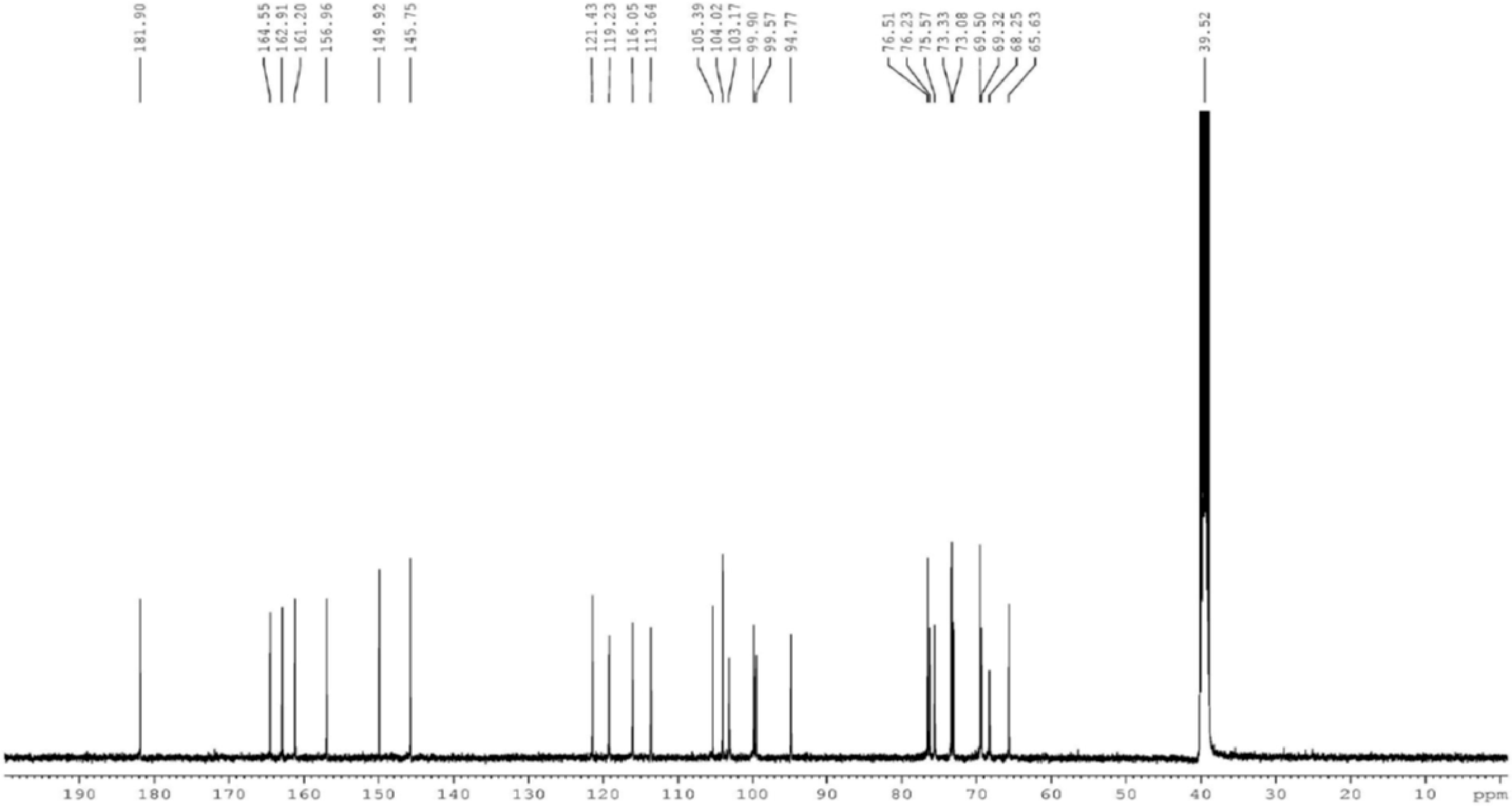


**Figure S4.** $^{13}C$ NMR spectrum (400.15 MHz, DMSO-$d_6$) of compound **9**.

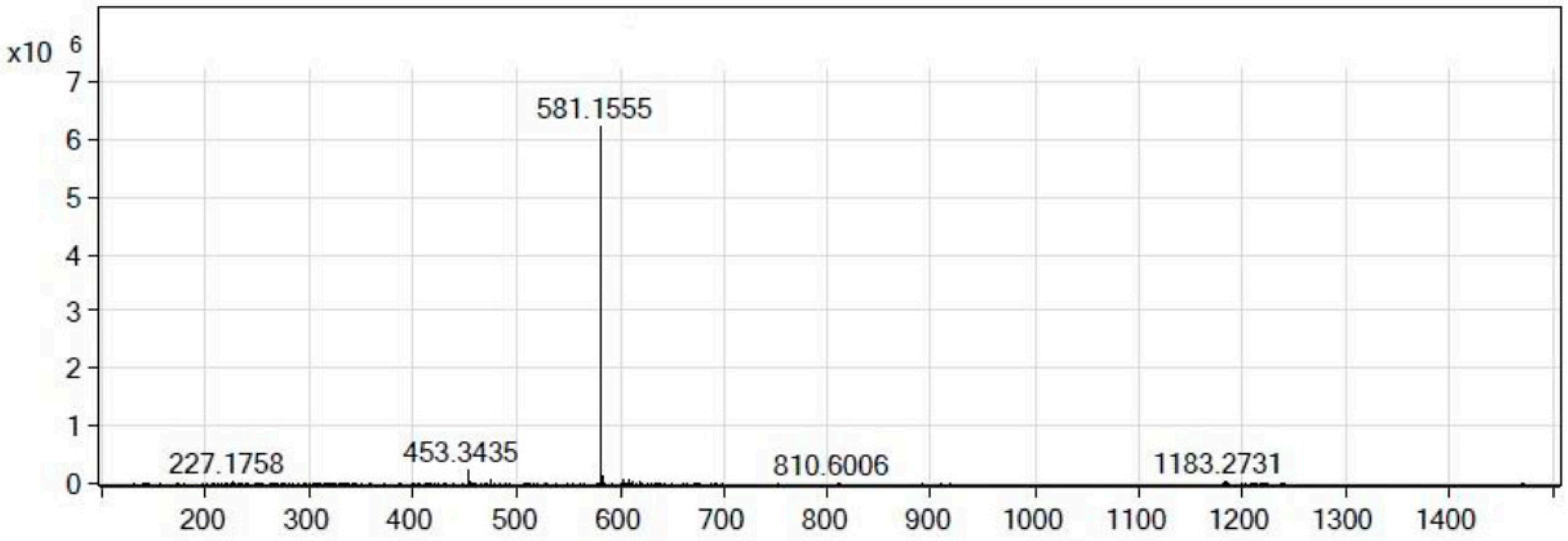


**Figure S5.** The MS spectrum of compound **9** in positive ion mode.

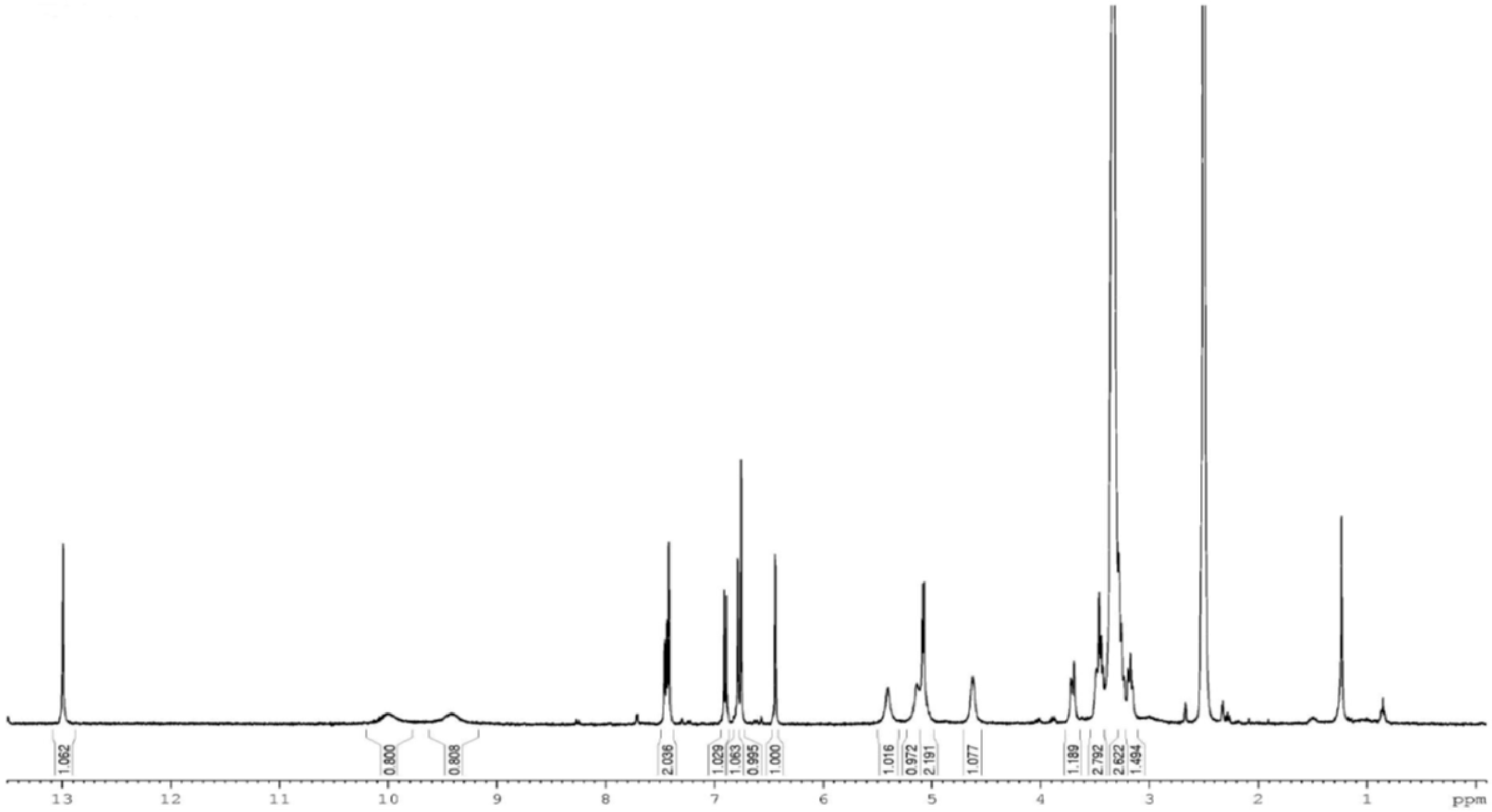


**Figure S6.** $^{1}H$ NMR spectrum (400.15 MHz, DMSO-$d_6$) of compound **12**.

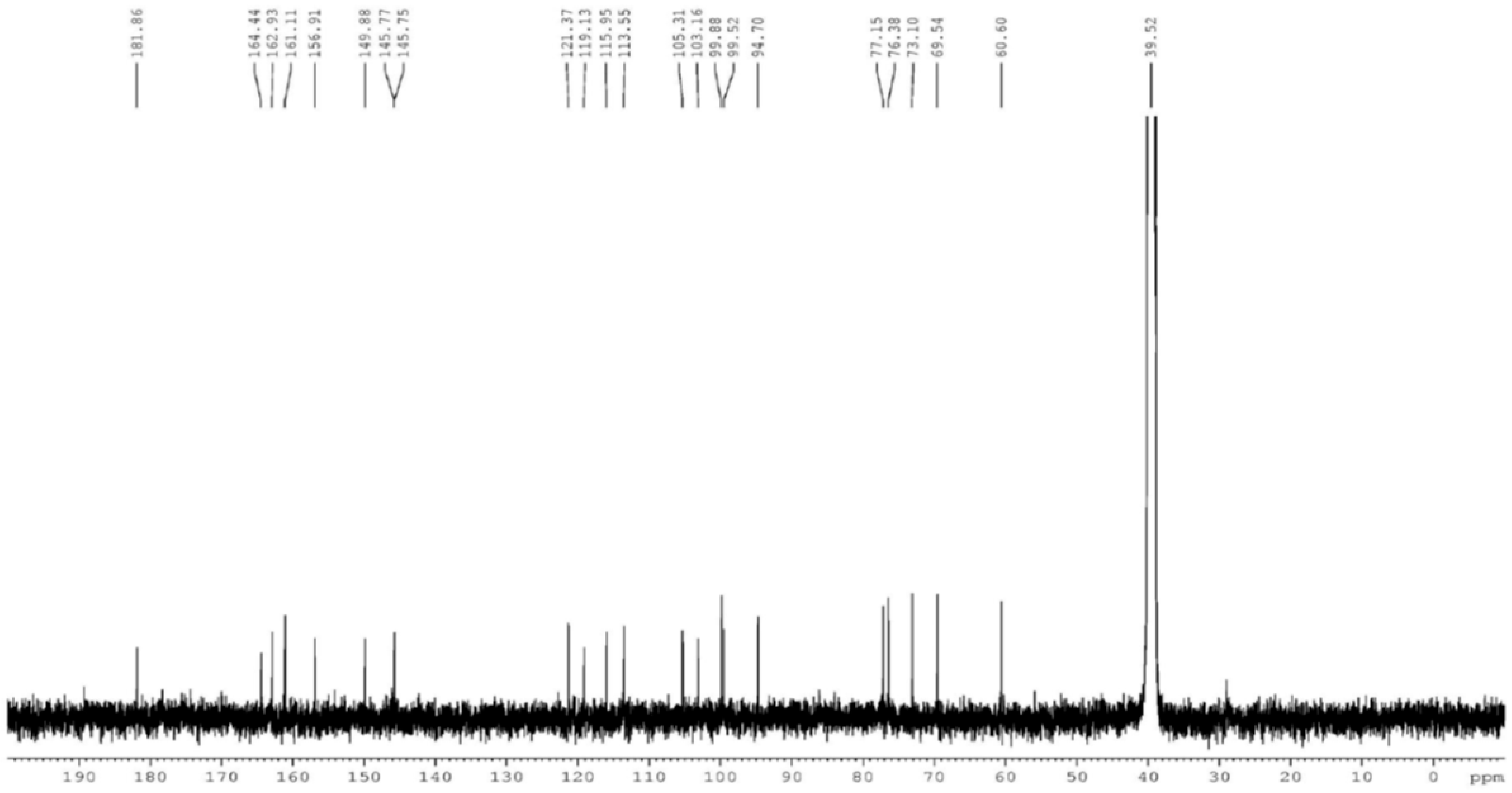


**Figure S7.** $^{13}$C NMR spectrum (400.15 MHz, DMSO-$d_6$) of compound **12**.

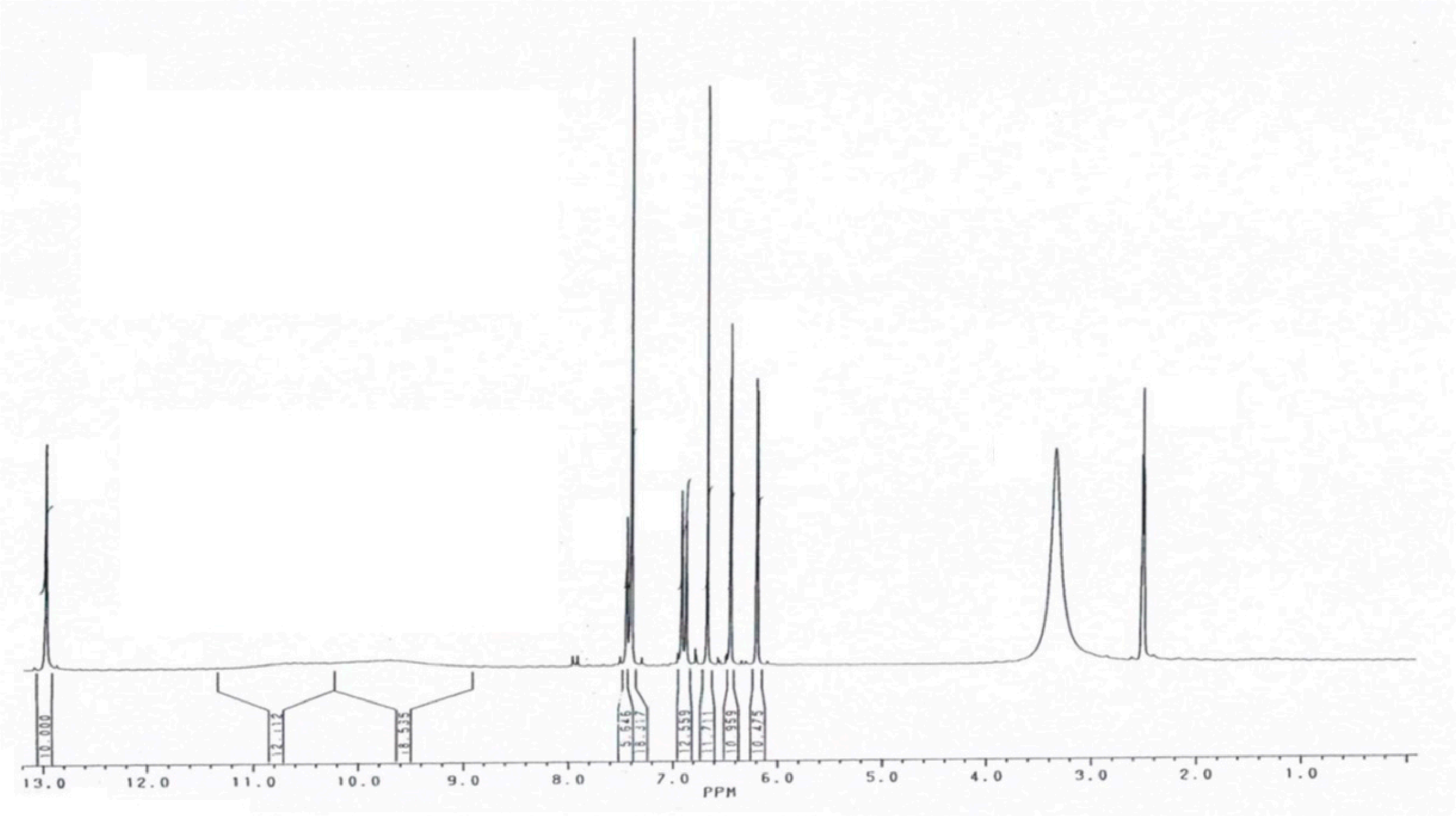


**Figure S8.** $^{1}H$ NMR spectrum (400.3 MHz, DMSO-$d_6$) of compound **22**.

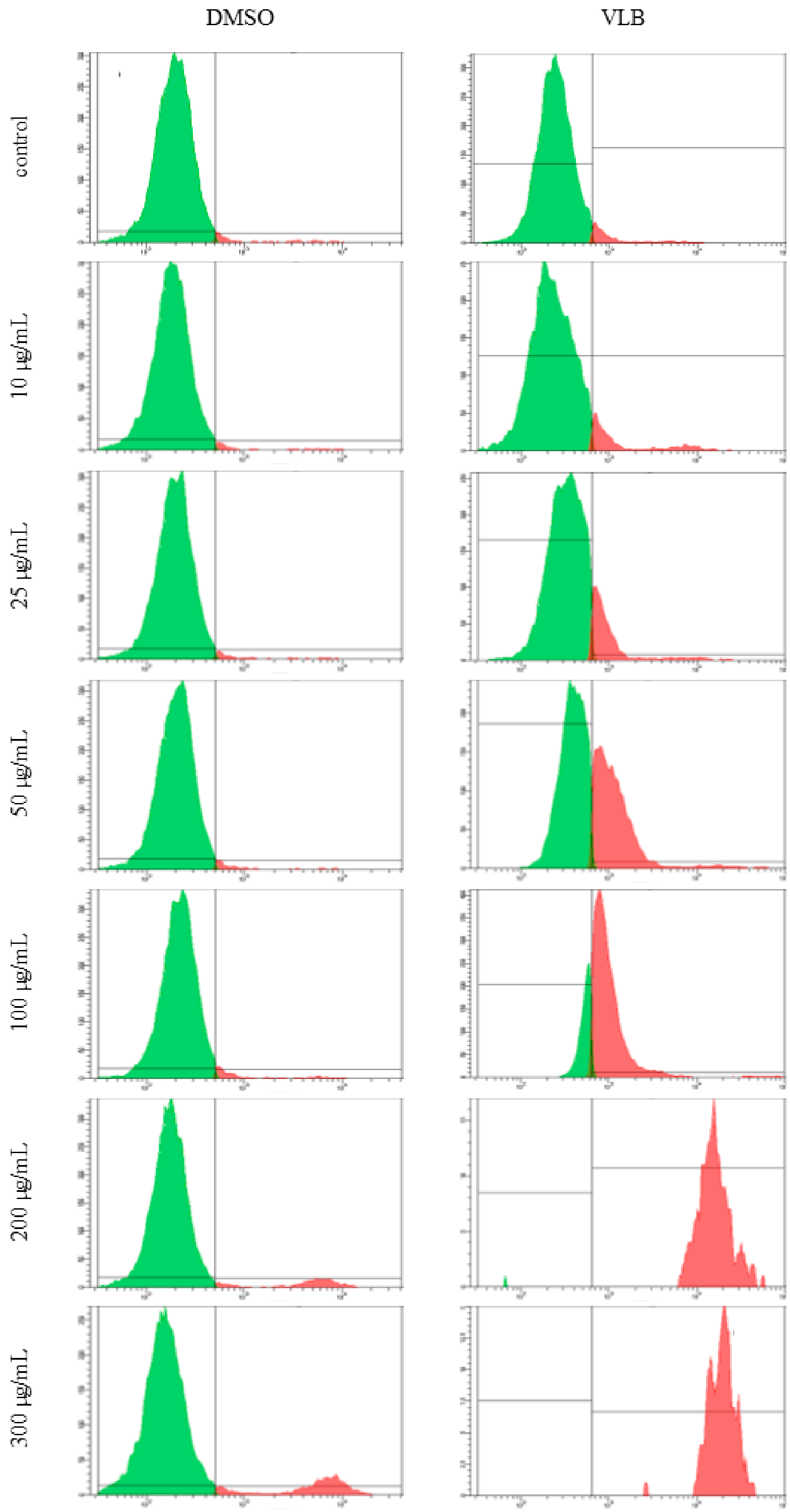


**Figure S9.** Flow cytometric analysis of cytotoxicity of C32 melanoma cells after 24 h of incubation with DMSO and VLB (10, 25, 50, 100, 200, and 300 µg/mL) comparable with untreated control by the Fixable Viability Stain assay.

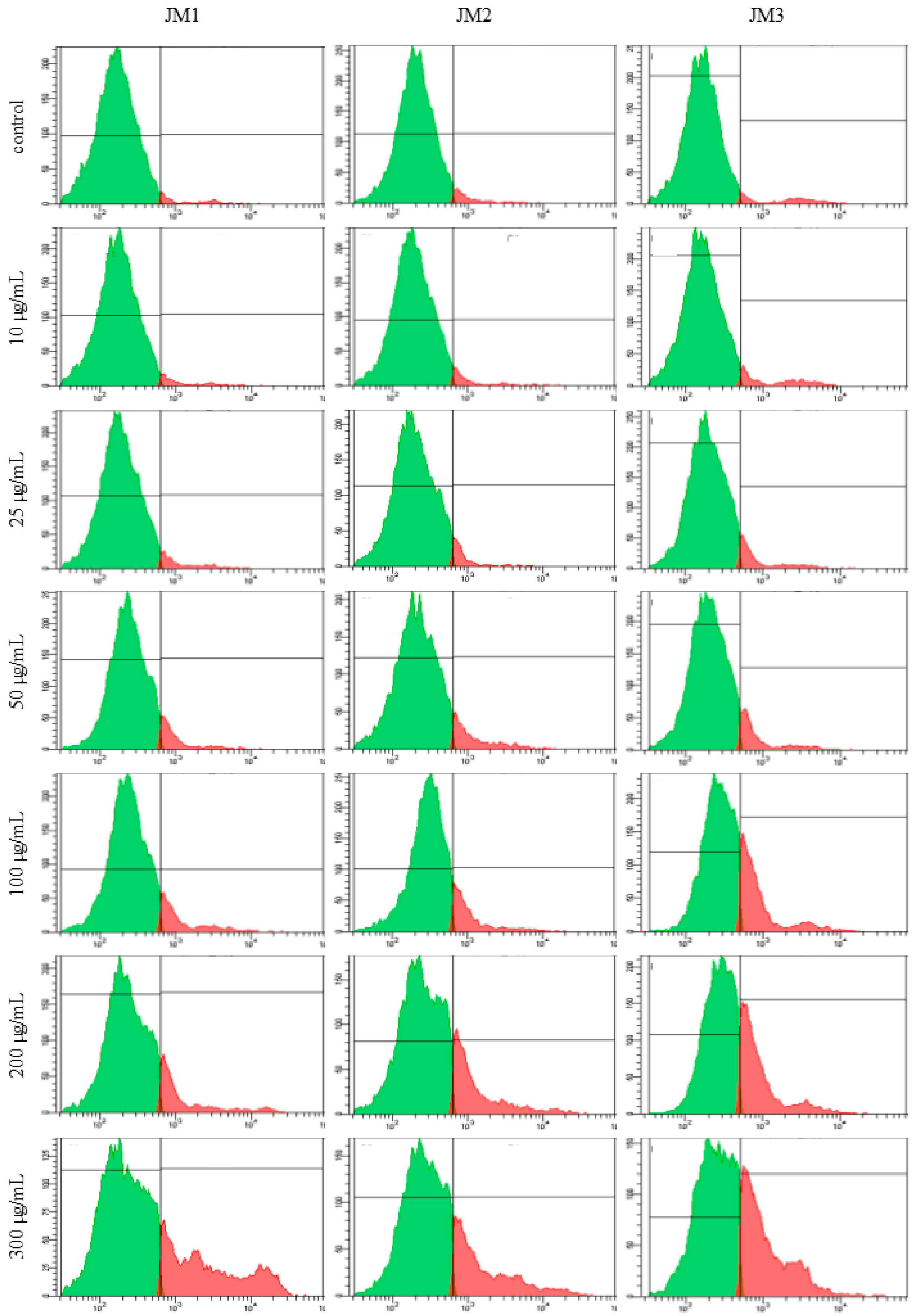


**Figure S10.** Flow cytometric analysis of cytotoxicity of C32 melanoma cells after 24 h of incubation with **JM1–JM3** (10, 25, 50, 100, 200, and 300 μg/mL) comparable with untreated control by the Fixable Viability Stain assay.

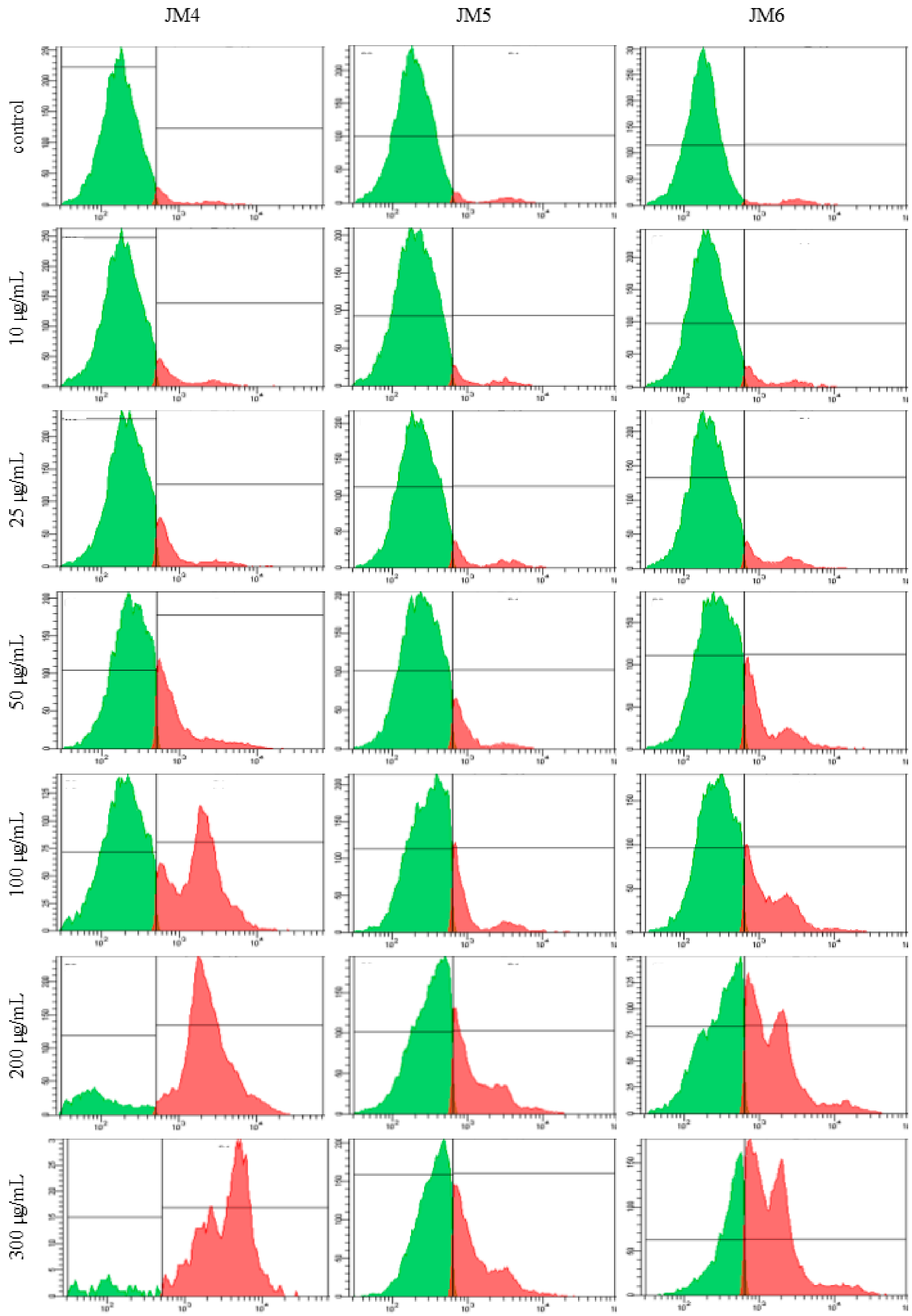


**Figure S11.** Flow cytometric analysis of cytotoxicity of C32 melanoma cells after 24 h of incubation with **JM4–JM6** (10, 25, 50, 100, 200, and 300 µg/mL) comparable with untreated control by the Fixable Viability Stain assay.

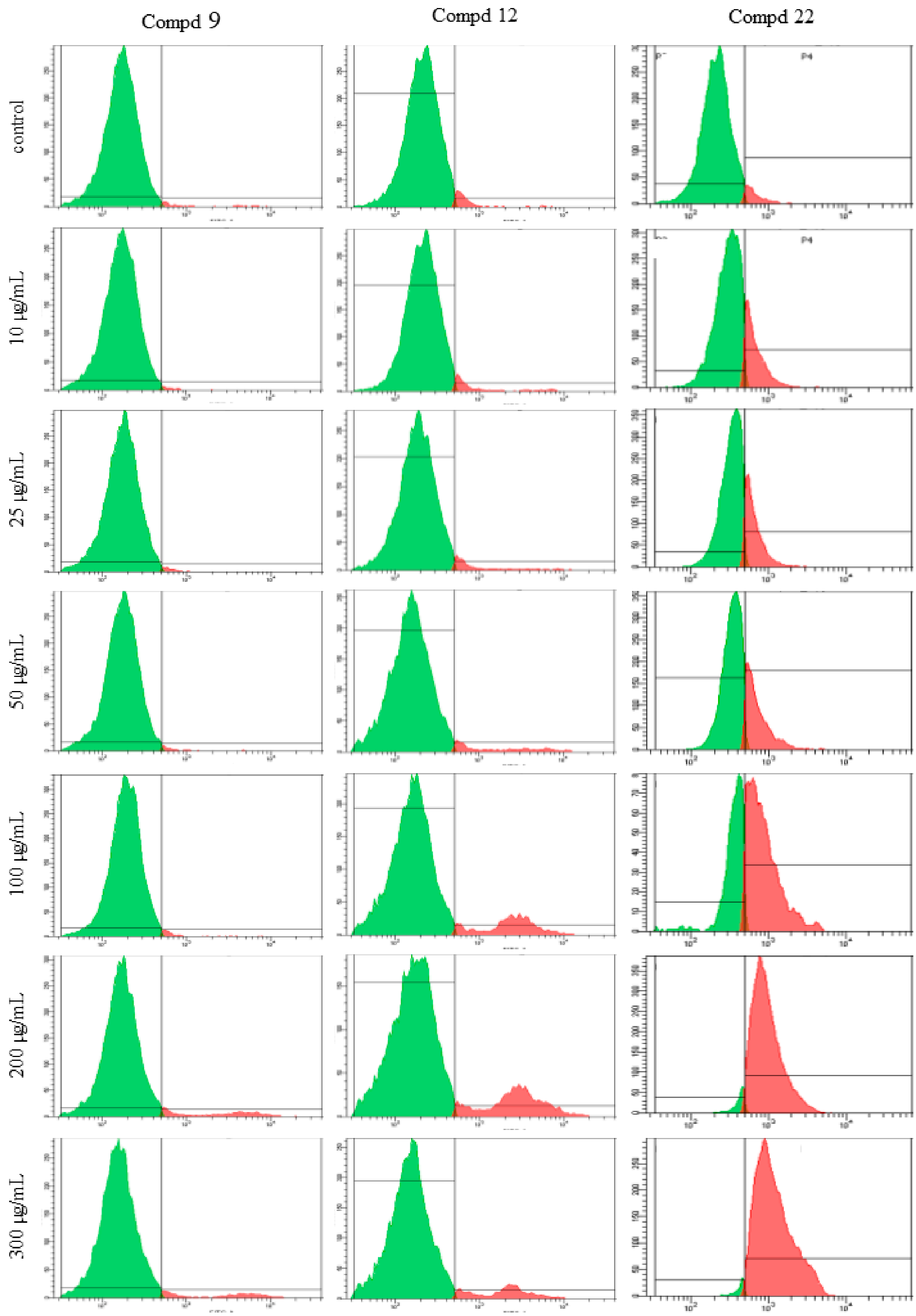


**Figure S12.** Flow cytometric analysis of cytotoxicity of C32 melanoma cells after 24 h of incubation with compound **9**, **12**, and **22** (10, 25, 50, 100, 200, and 300 µg/mL) comparable with untreated control by the Fixable Viability Stain assay.

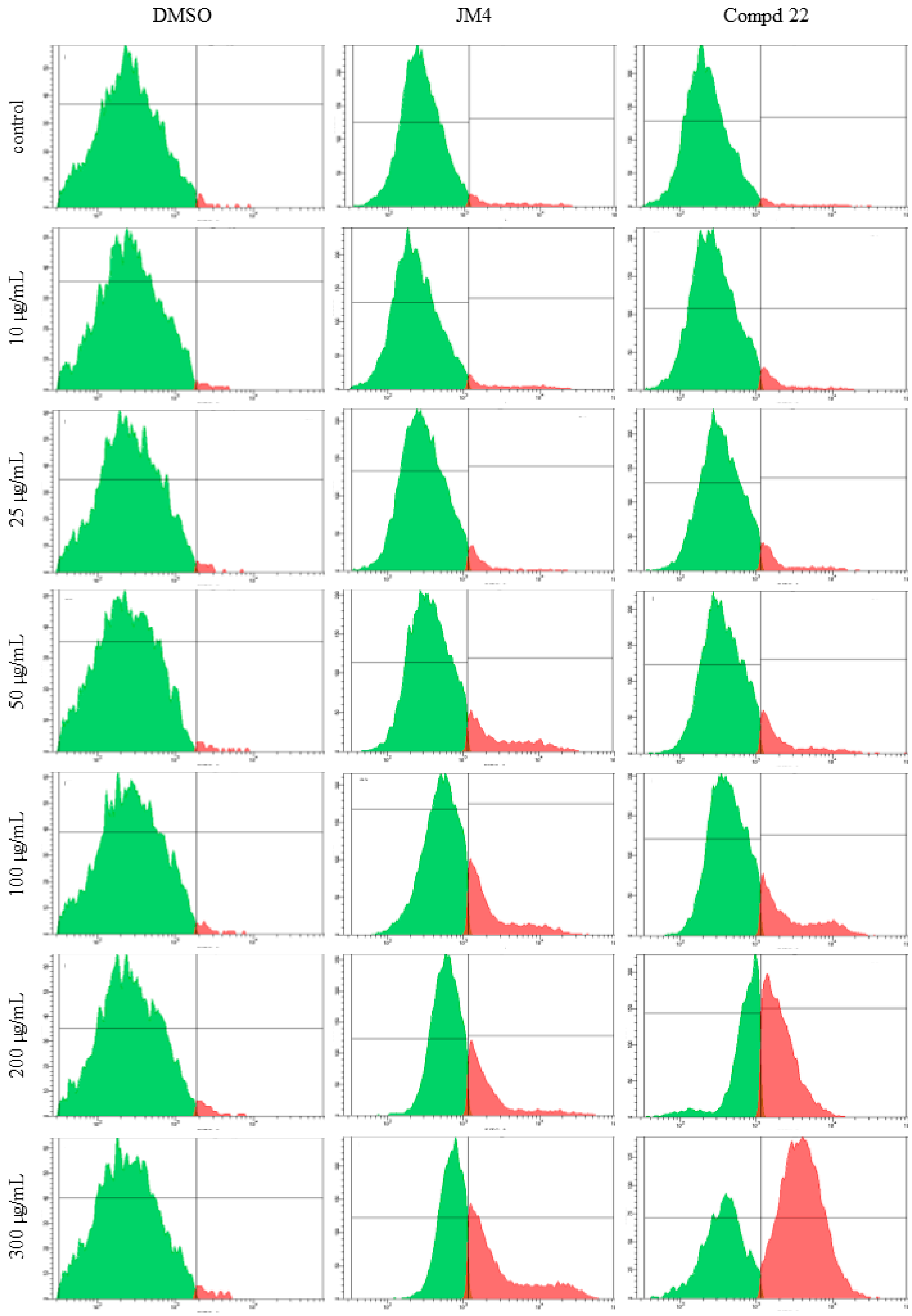


**Figure S13.** Flow cytometric analysis of cytotoxicity of normal human fibroblasts cells after 24 h of incubation with DMSO, **JM4**, and compound **22** (10, 25, 50, 100, 200, and 300 µg/mL) comparable with untreated control by the Fixable Viability Stain assay.
